# Stimuli-Responsive Multiacceptor Conjugated Polymers: Recent Trend and Future Direction

**DOI:** 10.1021/acspolymersau.4c00082

**Published:** 2025-01-21

**Authors:** Tamanna Pradhan, Dinesh Kumar Chelike, Debarshi Roy, Tanay Pramanik, Subrata Dolui

**Affiliations:** † Department of Chemistry, Indian Institute of Technology Kharagpur, Kharagpur, West Bengal 721302, India; ‡ Department of Chemistry, Rungta College of Engineering & Technology Bhilai, Kohka, Durg, Chhattisgarh 490024, India; § Jay FineChem Pvt. Ltd., Vapi, Gujarat 396191, India; ∥ Department of Chemistry, Institute of Engineering and Management, University of Engineering and Management Kolkata, University Area, Action area 3, Newtown, Kolkata 700160, India; ⊥ Graduate School of Advanced Science and Engineering, Hiroshima University, Kagamiyama 1-4-1, Higashihiroshima 739-8527, Japan

**Keywords:** Conjugated polymer, Stimuli-Response, RDRP, Biointerface, Optoelectronic, Antimicrobial, Sensor actuators, and Bioelectronics

## Abstract

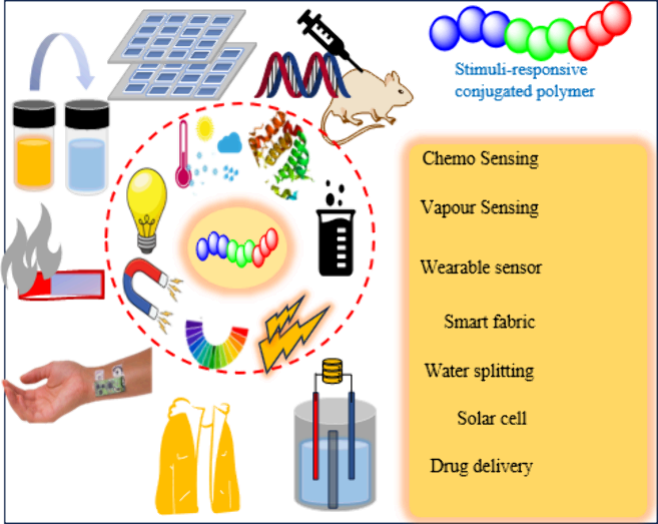

Apart from the visual effects, geometric shapes of materials play an important role in their engineering and biomedical applications. Responsive materials-based patient-specific anatomical models provide better insights into the structure and pathology. Polymers are by far the most utilized class of materials for advanced science and technology. Because of these properties, these polymers have been used as functional coatings, thermoplastics, biomedical materials, separators, and binders for Li-ion batteries, fuel cell membranes, piezoelectric devices, high-quality wires and cables, and so on. Reactive to stimuli because of their unusual electrical characteristics and adaptability, stimuli-responsive multiacceptor conjugated polymers have been a prominent focus of materials science study. These polymers combine several electron-accepting units inside their conjugated backbone, resulting in increased functionality and responsiveness to a variety of stimuli. The production, workings, and wide range of applications of stimuli-responsive multiacceptor conjugated polymers are the focus of this review.

## 1 Introduction

In this recent era the field of conjugated polymers has witnessed remarkable advancements, particularly in the development of stimuli-responsive multiacceptor conjugated polymers. These advanced materials have garnered significant attention due to their potential to revolutionize various fields, from electronics and photonics to environmental sensing and medicine. Stimuli-responsive polymers are designed to exhibit a change in their properties or behavior in response to external stimuli such as light, heat, pH, or chemical environments.^1−5^ The incorporation of multiple electron-acceptor units in these polymers further enhances their responsiveness and functionality. This multiacceptor approach allows for the tuning of electronic and optical properties, enabling sophisticated responses to a range of external triggers. Multistimuli responsive multiacceptor conjugated polymers are materials that have multiple electron-accepting units along their polymer chains. These polymers are of interest in the field of organic electronics, especially in the development of organic photovoltaic (OPV) devices, which are used to convert sunlight into electricity.^6−9^ There are several reasons why multiacceptor conjugated polymers are required and valuable in this context. Multiacceptor polymers can absorb a broader range of light wavelengths compared to single-acceptor polymers.^10,11^ In organic photovoltaic devices, the absorbed photons need to generate electron–hole pairs (excitons) that can be separated and then collected as electric current. Multiacceptor polymers can help extend the lifetime of photogenerated electrons, which is beneficial for increasing the chances of successful charge separation and, therefore, higher device performance.^12^ While multiacceptor conjugated polymers offer many advantages for OPV devices and other organic electronics applications, their design and synthesis can be more complex than single-acceptor polymers.^13^ Researchers continue to work on developing new materials and optimizing device architectures to fully harness the potential of multiacceptor conjugated polymers for renewable energy applications. Third-generation organic semiconductors have more intricate architectures, such as donor–acceptor (D-A) alternating copolymers along the main chains, and more atoms in the conjugated polymer main chains. In recent years, these materials have become recognized as stable semiconducting materials.^7,14−16^ Different techniques have been explored to improve the end functionalities and molecular architectures of conjugated polymers to make them suitable for various applications, as described in several reviews or book chapters. Heinze et al.^17^ for instance, detailed the significant advancements made in the field of CP electrochemistry during the past few years. Beginning with the electrochemical creation mechanism of CPs, it has been made clear that oligomerization happens in solution in front of the electrode and is best based on ionic (radicalic) species that go through successive “dimerization” phases. However, the topic of CPs’ multiacceptor system was left out. Schroot et al.^18^ reported building blocks to illustrate the simple assembly to Dn–P–Am architectures—which are effectively block copolymers with a single interjacent photosensitizer unit—were described as telechelic redox-active and conjugated polymers. Nezakati et al.^19^ reported the opportunity of conducting polymer and biomedical applications. CPs and their advantages and associated challenges, from synthesis to applications, were fully discussed but there is no multiacceptor system described. Inal et al.^20^ described conjugated polymers in bioelectronics but multiacceptor system in CPs were not covered. All these reviews mainly focused on the synthesis of conjugated polymers via different strategies and their potential applications. However, there is no comprehensive review in the past 10 years (and before) Notably, multistimuli responsive multiacceptor in conjugated polymers have never been reviewed (in contrast to other conjugated polymers) which have been reviewed extensively. Moreover, none of the reviews mentioned in this section focused on the aspects and uniqueness of stimuli-responsive multiacceptor in conjugated polymers which may introduce smart properties in such conjugated polymers. Furthermore, the development of polymer analogous reactions,^21^ including postpolymerization modifications^22^ via ‘‘click” chemistry^23^ have emerged as a highly efficient tool to introduce specific functionality to the polymer. In the last couple of decades, stimuli-responsive polymeric materials, in its solid/solution/gel state, capable of responding to various chemical/physical/biochemical stimuli,^24^ including pH,^25,26^ temperature,^27^ ionic strength,^28^ solvents,^29^ gases,^30^ and redox,^31^ were prepared for applications in materials and biomedical applications.^32^ During the last couple of decades, extensive research and development have taken place in the field of conjugated polymers via various polymerization strategies. However, to the best of our knowledge, there have been no review articles on multiacceptor in conjugated polymers. Multiacceptor conjugated polymers offer a promising avenue for improving the performance and efficiency of organic electronic devices, particularly in the realm of renewable energy conversion. Their ability to enhance light absorption, charge separation, and energy utilization makes them a subject of significant interest for researchers and industry professionals seeking to advance green energy technologies. “Multi-stimuli responsive Multi-Acceptor Conjugated Polymers for Organic Electronics*”* addresses a question at the interface of macromolecular Science, Material Science, and chemistry which has the potential to impact High Tech applications mentioned above in the coming century. Such types of multiacceptor conjugated polymers combine the unique features of polyenes with those of the sequence-controlled macromolecules. Thus, it brings the best of two worlds together, enabling the design of interesting and smart materials for emerging applications to make us future-ready.

## 2 Structure and Synthesis of Multiacceptor Conjugated Polymers

**Figure 1:**
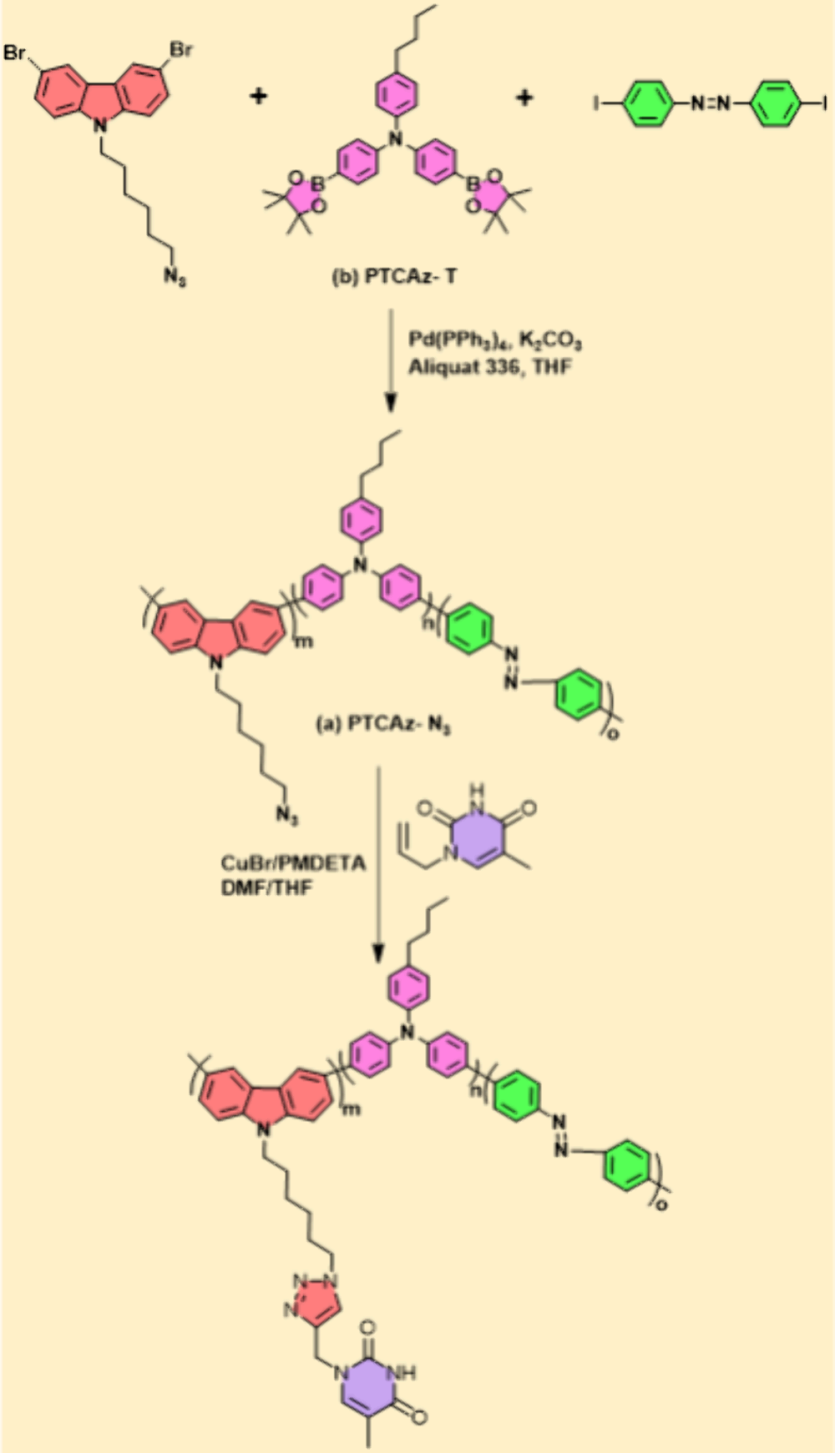
Preparation of the conjugated polymers (a) PTCAz-N_3_ (through Suzuki coupling polymerization) and (b) PTCAz-T (through click reactions of PTCAz-N_3_ with PT). Reproduced with permission of ref (50). Copyright 2018 Royal Society of Chemistry.

The same conjugated backbone structure has been used to design and synthesize a vast number of conjugated polyeletrolytes (CPEs) during the last few decades. All the CPEs can be categorized based on their structure functionalities. The existing procedures includes Suzuki,^33−36^ Heck^37−40^ and Sonogashira,^41−44^ Wessling reaction,^45,46^ photopolymerization reaction,^47−49^ and FeCl_3_ oxidative polymerization for the synthesis of different CPEs. In 2018 Huang et al.^33^ proposed a simple approach using Suzuki coupling polymerization and click reactions to create conjugated copolymers (PTCAz-T) functionalized with thymine (T) and azobenzene (bioinspired) as depicted in (Figure 1). After completing the click reactions, the glass transition temperature of PTCAz-T is higher than that of its precursor, the T-free PTCAz-N_3_ conjugated copolymer. This is because the strong T–T interactions limited molecular motion, reduced free volume, and produced a more stable morphology. The conjugated polymer presenting the T units exhibited an amorphous phase behavior without macro-phase separation due to the presence of azobenzene units in its main chain. This property enabled the preparation of homogeneous photocontrollable thin films that exhibited stimuli-responsive behavior through photoinduced trans-to-cis isomerization.

## 3 Single-Stimuli-Responsive Conjugated Polymers

**Figure 2:**
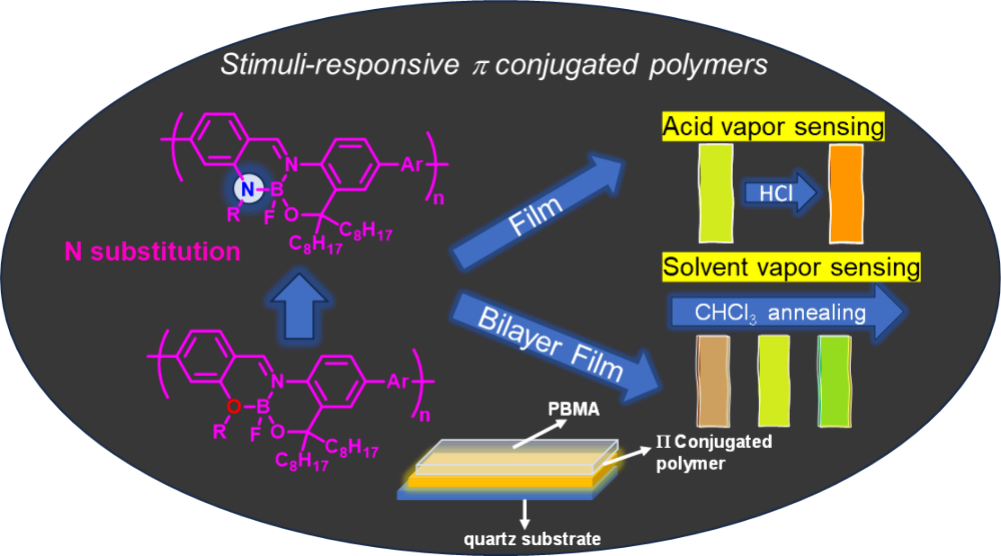
Stimuli-responsive π-conjugated polymers showing solid-state emission based on boron-fused azomethine complexes with NNO-tridentate ligands. Adopted with permission from ref (51). Copyright 2023 American Chemical Society.

A recent advancement in this field is integrating many stimuli into a single polymer in order to achieve improved specificity. In order to have access to the most recent advancements in stimulus-responsive polymers, the objective of this review is to integrate multiple stimuli. The combinations of different stimuli that can trigger it are examined, as well as chemical structures that can cause it. In 2023 Tanaka et al.^51^ reported boron-fused azomethine (BAm) structure with NNO-tridentate ligands, resulting in stimuli-responsive luminous π-conjugated polymers that show promise for use in film-type sensors. The unbound lone-pair of the nitrogen atom on the BAm unit provide the polymers their acid-responsive characteristics. Under acid vapor, the polymer films exhibited redshifts in emission wavelengths; these reactions were reversible and associated with deprotonation. Additionally, because of the film’s sensitivity to the environment, distinct luminous color shifts were noticed as represented in (Figure 2).

### 3.1 pH-Responsive Conjugated Polymer

**Figure 3:**
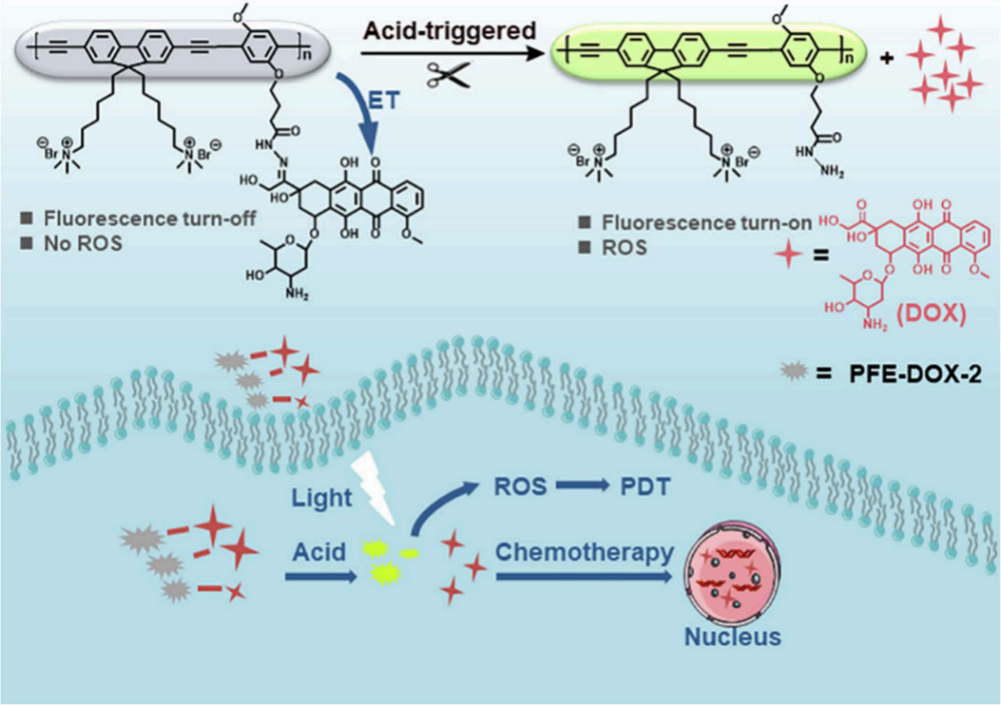
Schematic diagram of pH-responsive drug release and the mechanism of synergistic chemo-/PDT antitumor therapy. Adopted with permission under Creative Commons CC BY 4.0 license from ref (52). Copyright 2023 The Author(s).

A pH-responsive conjugated polymer is a type of polymer that can undergo a change in its optical, electronic, or chemical properties in response to changes in pH.^52,53^ These materials combine the characteristics of conjugated polymers—such as extended π-conjugation, which allows for electrical conductivity and light absorption—with pH-sensitivity. The pH sensitivity usually comes from functional groups incorporated into the polymer structure, such as carboxyl, amine, or sulfonic acid groups. These groups can ionize or deionize in response to changes in pH, leading to changes in the polymer’s electronic properties, solubility, or physical structure. Here, a conjugated polymer-based pH-responsive drug delivery system was developed for efficient synergistic chemo-/PDT antitumor therapy as displayed in (Figure 3). In PFE-DOX-2, an anticancer medication called DOX is covalently bonded to the polymer’s side chain by an acid-sensitive acyl hydrazone bond. This can enhance the durability of the drug delivery system and stop early drug leakage.

### 3.2 Temperature-Responsive Conjugated Polymer

**Figure 4:**
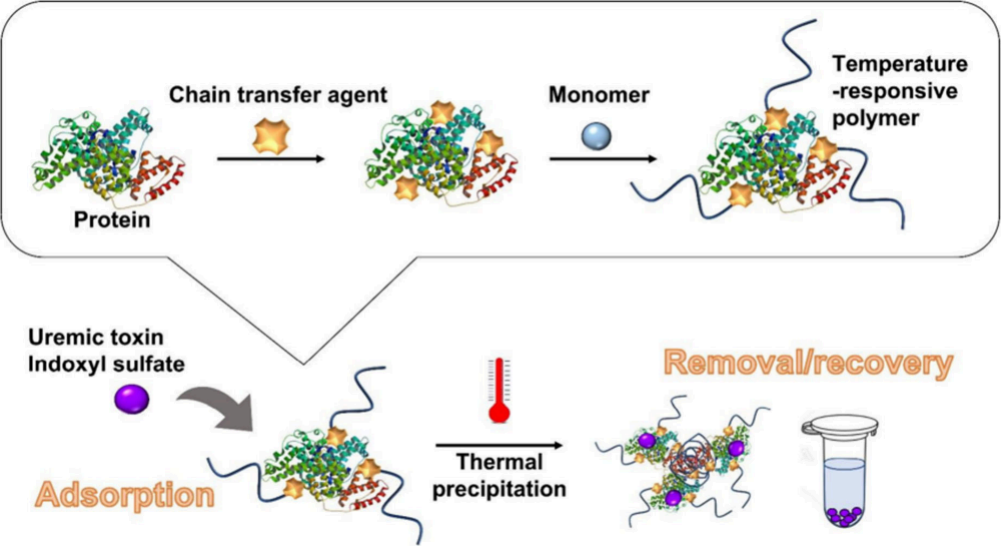
Schematic of the proposed thermally responsive polymer–protein conjugate system. First, protein-PNIPAAm is prepared by “grafting from” method. Next, the conjugate is allowed to capture indoxyl sulfate (IS), one of the uremic toxins. Finally, the captured IS is recovered by thermal precipitation. Adopted with permission under Creative Commons CC BY 4.0 license from ref (58). Copyright 2022 The Author(s).

Temperature-responsive conjugated polymers are a fascinating class of polymers that undergoes changes in their properties depending on temperature. With the combination of electronic and optical characteristics of conjugated systems with the dynamic responsiveness of thermoresponsive materials.^54−57^ By incorporating temperature-sensitive units, researchers have developed polymers that not only retain their inherent conjugated characteristics but also exhibit controlled changes in response to temperature fluctuations. This dual functionality enhances their potential in adaptive and smart materials. Yoshihara et al.^58^ reported in 2022 that a temperature-responsive polymer–protein combination was created by grafting bovine serum albumin (BSA) with a chain transfer agent (CTA) utilizing the “grafting from” approach. Poly(*N*-isopropylacrylamide) (PNIPAAm) was synthesized by reversible addition–fragmentation chain transfer polymerization, (RAFT) with the BSA-CTA serving as the starting point. Around the LCST, PFBT-gPA experiences a temperature shift that causes a reversible phase transition as depicted in (Figure 4). Additional research shows that PFBT-gPA may withstand photobleaching in an aqueous solution without the need for additional antioxidants. A temperature change can adjust the photosensitizing capacity.

### 3.3 Light-Responsive Conjugated Polymer

**Figure 5:**
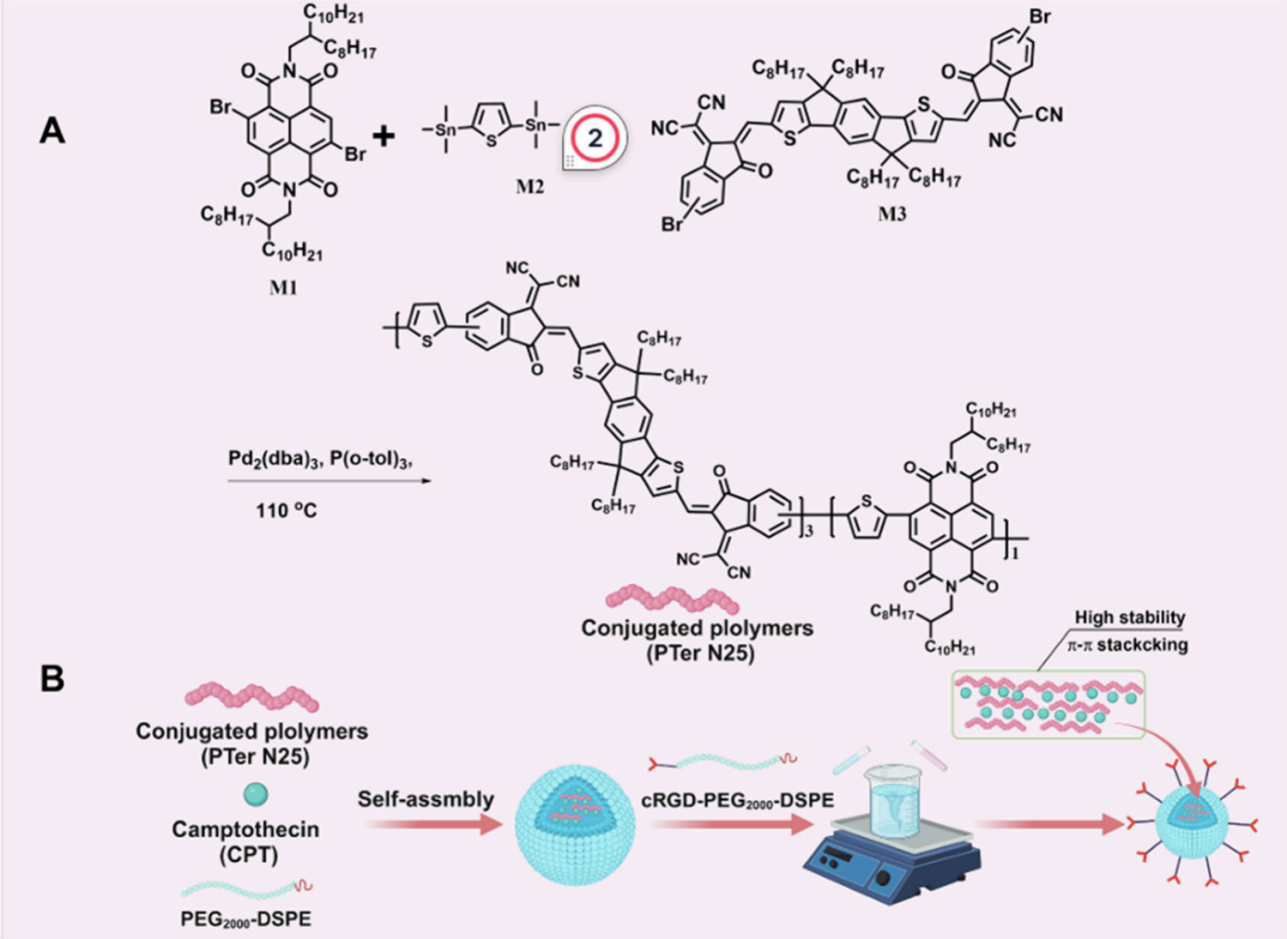
Conjugated polymer (PTer N25, chemical structure shown). Adopted with permission from ref (64). Copyright 2024 The Authors.

Conjugated polymers are known for their ability to conduct electricity and exhibit unique optical properties due to their extended π-conjugation.^59−63^ By incorporating light-sensitive units or using light-responsive mechanisms, these polymers can be engineered to change their behavior—such as conductivity, fluorescence, or structure—when exposed to light.^64^ This light-responsiveness enables the development of advanced materials with adaptive functionalities for diverse applications. Many light-responsive polymers incorporate photoisomerizable moieties like azobenzene or spiropyran. For instance, azobenzene undergoes reversible trans–cis isomerization upon UV and visible light exposure, leading to changes in the polymer’s optical and physical properties. This process is used to induce changes in polymer morphology or properties. Three different forms of switchable and monodisperse conjugated polymer particles were synthesized, as reported by Anwar et al.^65^ in 2013. Fluorene-*alt*-azobenzene polymer particles are created by Suzuki-Miyaura dispersion polymerization. Particle size is easily tunable, and fluorescence intensifies when trans-to-cis flipping occurs. In 2024 Zeng et al.^64^ reported on the development of a conjugated polymer (PTer N25, (Figure 5) as a nanocarrier drug delivery system (DDS) for camptothecin (CPT), based on indenyl dithiophene-diketopyrrolopyrrole (IDTIC) and naphthalimide (NDI).

### 3.4 Redox-Responsive Conjugated Polymer

**Figure 6:**
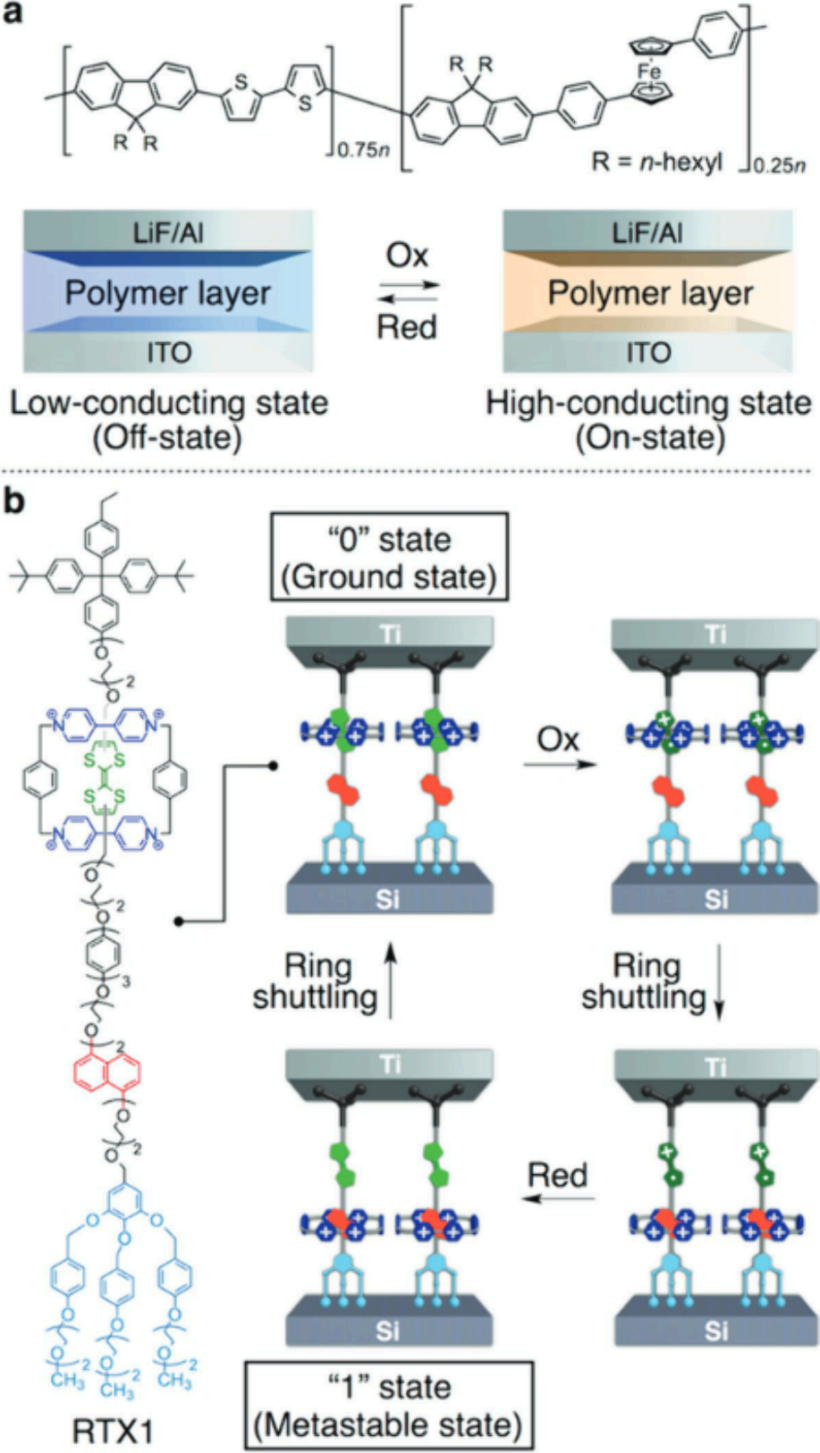
Redox-modulable electronic memory. (a) Schematic representation of an electrochemically modulable nonvolatile resistive memory device consisting of a spin-coated film of an Fc-doped π-conjugated polymer (50–60 nm thick) sandwiched between indium tin oxide (ITO) and LiF/Al electrodes. Adopted with permission from ref (72). Copyright 2007 American Chemical Society. (b) Schematic representation of the molecular structure of bistable [2]rotaxane RTX1 for dynamic random-access memory (DRAM) in which reversible electrochemical oxidation and reduction of RTX1 allow information writing and erasing. Adopted with permission from ref (71). Copyright 2007 Springer Nature Limited.

Redox-responsive materials can undergo changes in their chemical structure or properties when they gain or lose electrons.^66−69^ This can involve changes in their oxidation state, which can alter their color, conductivity, or other properties. An electrochemically tunable nonvolatile resistive memory based on a π-conjugated polymer comprising fluorene, bithiophene, and Fc units was described by Choi et al.^70^ The most recent iterations of molecular shuttles, NDI-[79] and RV^2+^-based redox-responsive daisy-chain rotaxanes, were reported by Choi^70^ and Green^71^ in 2014 (Figure 6**a,b**). These molecular shuttles take on a cyclic dimer form, where two cross-threaded molecules of a monomer containing ring and axis moieties are self-complementary as shown in (Figure 6b).

### 3.5 Chemoresponsive Conjugated Polymer

**Figure 7:**
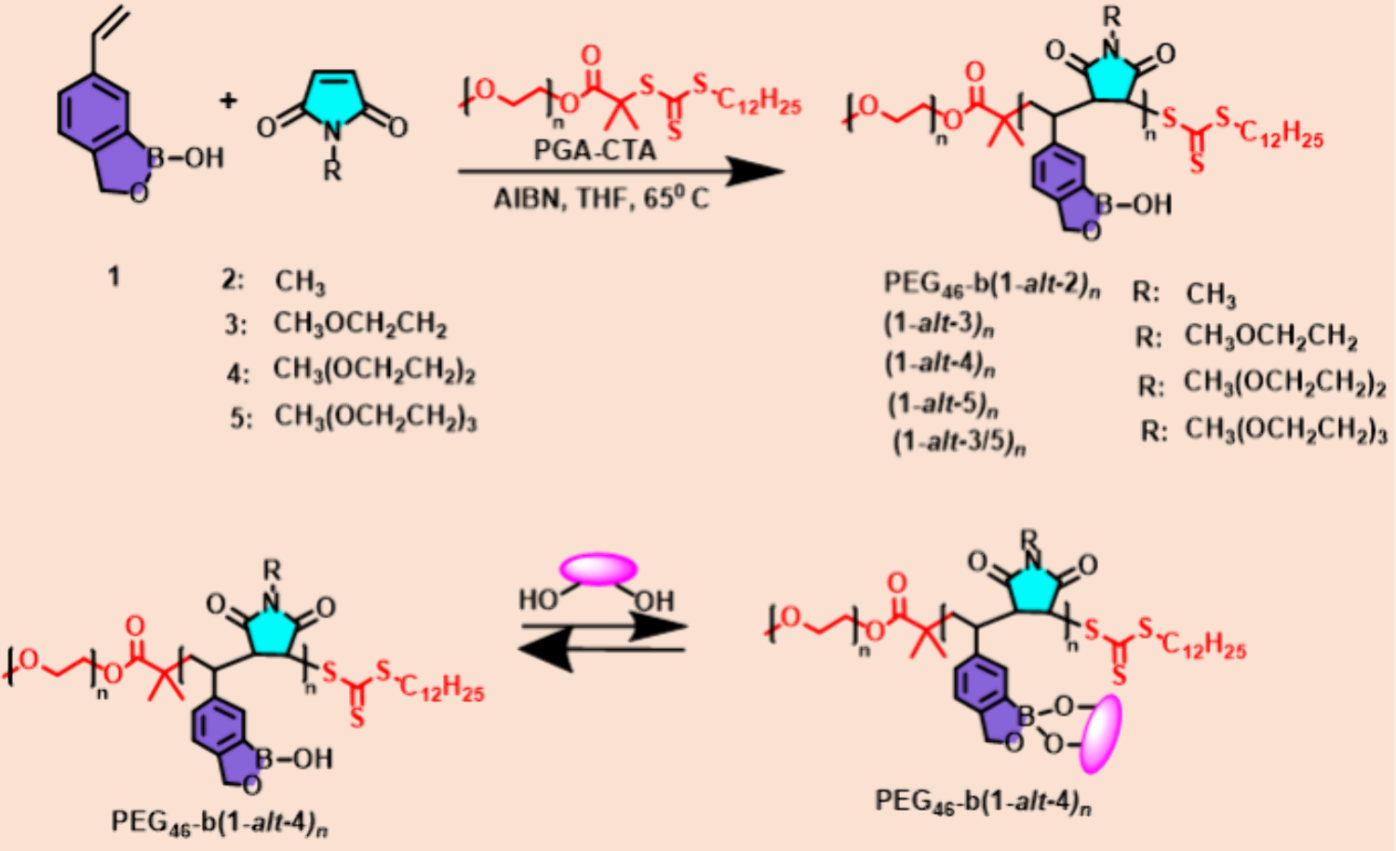
Synthesis of sequence-specific block copolymers of styrene boroxole and *N*-functionalized maleimide by RAFT polymerization and a schematic representation of sugar-responsive behavior of block copolymers in water. Adopted with permission from ref (82). Copyright 2012 American Chemical Society.

Chemoresponsive polymer materials are those whose structures alter in response to chemical agents acting as external stimuli.^73,74^ This behavior can be demonstrated by changes in the material’s volume form and other properties, such as optical and mechanical. Chemoreceptive polymer materials can often be divided into multiple subclasses. Most systems that have been reported are susceptible to pH changes,^75,76^ the presence of inorganic or organic anions,^77,78^ cations,^79^ or enzymes.^80,81^ Chemoreceptive materials also consist largely of redox-based systems. In 2012 Kim et al.^82^ reported a novel class of sugar-responsive block polymers based on a sequence-specific copolymer of N-functionalized maleimide and styreneboroxole as displayed in (Figure 7). To produce the stimuli-responsive block polymer, this pair of monomers underwent RAFT copolymerization in the presence of a poly(ethylene glycol)-chain transfer agent. This guaranteed that the glucose receptor boroxole alternated with a nonresponsive solubilizing group throughout the sugar-responsive polymer block. The solubility-switching boroxole moieties in the membrane-forming block were linked by hydrophilic solubilizing groups, allowing the polymersomes of the resulting block copolymers to disassemble at a lower glucose concentration, close to the physiologically relevant concentration at neutral pH.

### 3.6 Thermo- and pH-Responsive Conjugated Polymer

**Figure 8:**
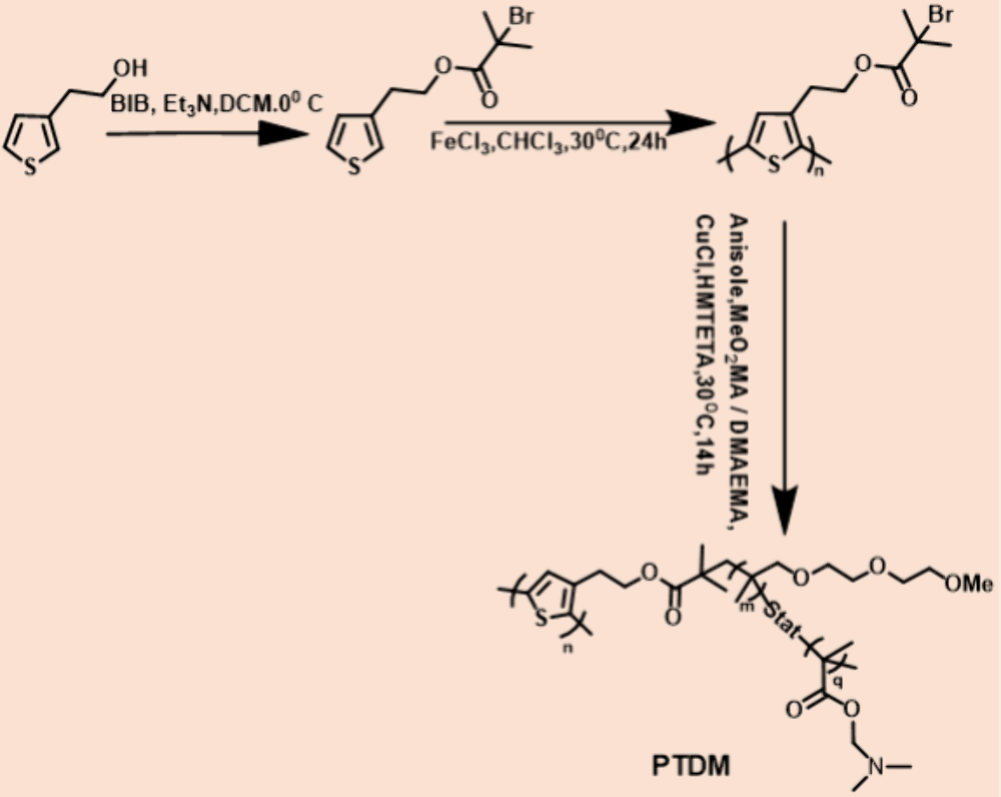
Synthesis procedure adopted for the preparation of the PTDM graft copolymer. Adopted with permission from ref (85). Copyright 2013 Wiley Periodicals, Inc.

The production of temperature-responsive, water-soluble PT-based graft copolymers using poly(*N*-isopropylacrylamide) (PNIPAM) as grafted chains has been described by Balamurugan et al.^83^ These copolymers have demonstrated notable water solubility and thermoresponsivity within the physiological temperature range (∼37 °C). Using poly(N,N-dimethyl aminoethyl methacrylate) (PDMAEMA) as the grafted chains, Wang et al.^84^ reported a water-soluble pH responsive PT based graft copolymer in a different attempt. This research group has recently published a paper on the grafting of oligoethylene glycolmethyl ether methacrylate (OEGMA) and poly(diethylene glycolmethyl ether methacrylate) (PMeO_2_MA) on a polythiophene backbone, demonstrating variable thermoresponsive and notable water solubility.^85^ In 2013 Das et al.^85^ reported that a water-soluble thermoresponsive graft copolymer with pH responsiveness. The 2,5-poly(3-[1-ethyl-2-(2-bromoisobutyrate)]) thiophene macroinitiator (PTI) was created by attaching the initiator moiety (2-bromoisobutyryl bromide) to 3-thiophene ethanol and polymerizing with ferric chloride. The polymerization was carried out at 30 °C utilizing copper-based atom transfer radical polymerization (ATRP) using a mixture of various compositions of diethylene glycol methyl ether methacrylate (MeO_2_MA) and N,N-dimethyl aminoethyl methacrylate (DMAEMA), as shown in (Figure 8.)

### 3.7 Thermo- and Light-Responsive Conjugated Polymer

**Figure 9:**
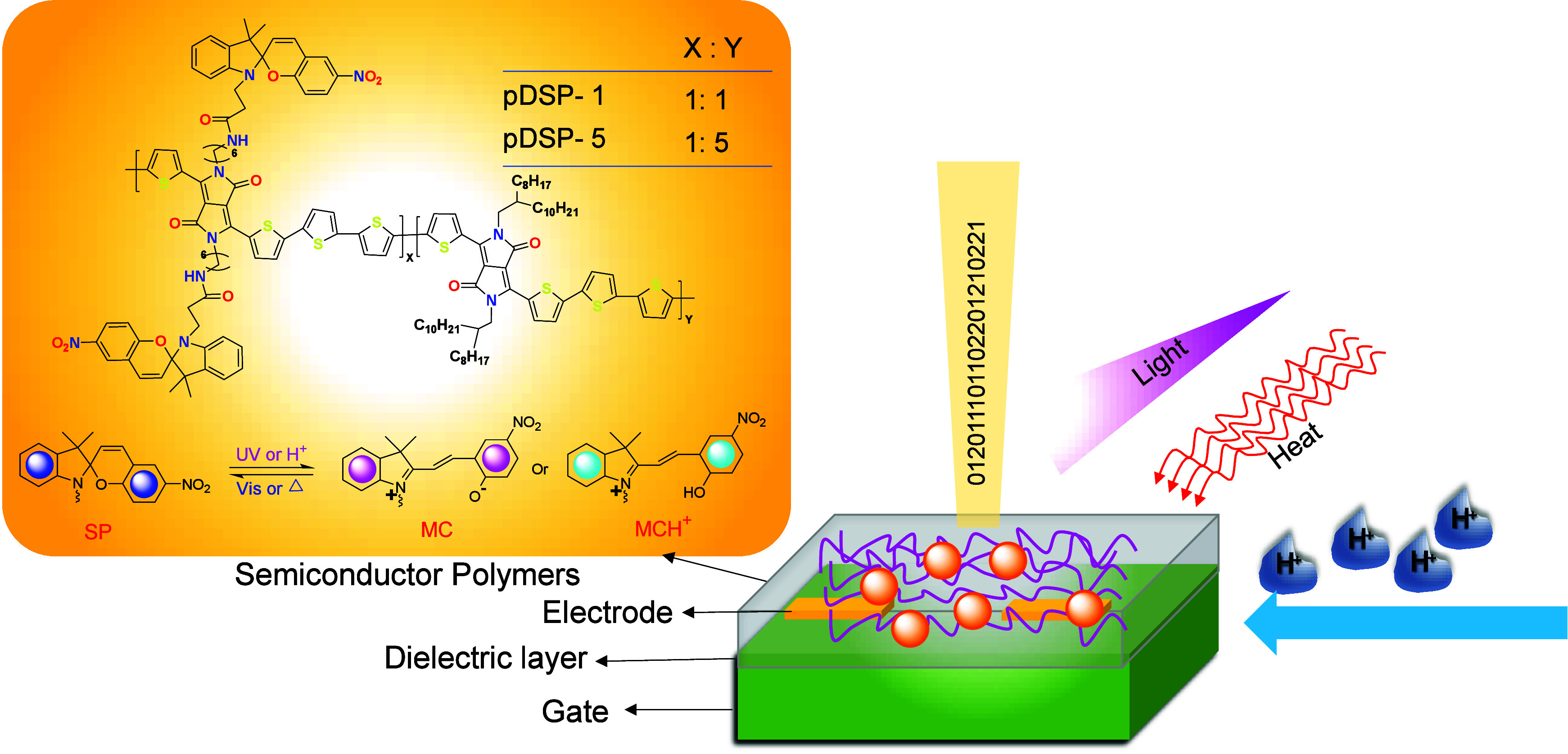
Chemical structure of pDSP-1 and the schematic representation of the multistimuli-responsive FET architecture. For comparative mechanistic studies, pDSP-5, containing different contents of SP groups in the side chains, was also prepared. Adopted with permission from ref (5). Copyright 2019 Chinese Chemical Society.

Organic and polymeric semiconductors with high charge mobilities have developed rapidly in the recent several decades. Improved conjugated molecules and polymers have been devised, synthesized, and novel techniques for device manufacturing have been put forth in an effort to boost organic semiconductors’ semiconducting capabilities.^86−88^ Thin-film field-effect transistors (FETs) derived from organic and polymeric semiconductors have demonstrated promising prospects in wearable electronics, flexible displays, and memory devices (Figure 9).^89^ Ma et al.^5^ disclosed in 2018 a novel method of creating polymeric semiconductors that respond to many stimuli by including spiropyran (SP) groups into the side chains of the polymers. The conjugated donor–acceptor (D–A) polymer based on diketopyrrolo-pyrroles (DPP) that contains SP units in the side chains (pDSP-1) of the resulting FETs’ semiconducting performances could be reversibly adjusted by heating and treating with acid, by heating and treating with UV light, or by heating and treating with visible and UV light.

## 4 Multistimuli-Responsive Conjugated Polymer

**Figure 10:**
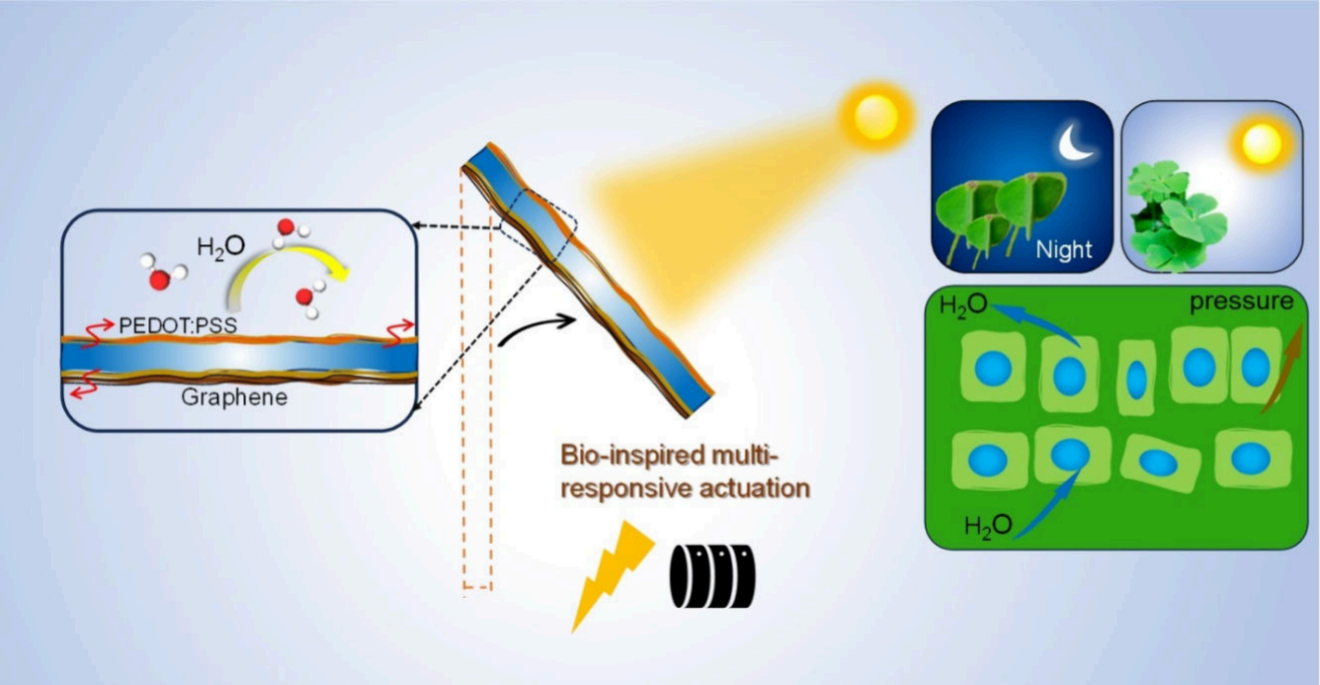
Enable multi-stimuli-responsive biomimetic actuation with asymmetric design of graphene-conjugated conductive polymer gradient film. Adopted with permission from ref (92). Copyright 2023 American Chemical Society.

The selected examples of polymers that react to two stimuli effectively highlight the potential of combining several inputs into one polymer as illustrated in (Figure 10). However, there has been a recent upsurge in the study of polymers that respond to triple stimuli—a stimulus that entails the addition of a second stimulus. The polymer’s increasing complexity makes it advantageous to add one more stimulus since it can improve precision, widen the switching window, or even change the switching conditions.^5,90,91^ Multistimuli responsive polymers offer more functionality and precise control than single stimuli responsive polymers do. Because of the polymer’s increased complexity, adding more stimuli can improve precision and expand the switching window and circumstances. Using a straightforward drop casting technique, graphene-conjugated poly(3,4-ethylenedioxythiophene): poly(styrenesulfonate) (PEDOT:PSS) gradient films have been used to create multiresponsive actuators with an asymmetric design.^92^ The hygroscopic expansion feature of PEDOT:PSS and the gradient distribution of graphene sheets within the film, which mimics the hierarchical swelling tissues of certain plants in nature, are credited with the biomimetic actuation. Actuators made of graphene conjugated PE-DOT:PSS (GCP:PSS) can bend in both directions in response to a variety of stimuli, including moisture, organic vapor, electrothermal heating, and photothermal heating.

### 4.1 Synthesis of Stimuli-Responsive Conjugated Polymers

#### 4.1.1 Synthesis via Reversible Addition–Fragmentation Chain Transfer Polymerization

**Figure 11:**
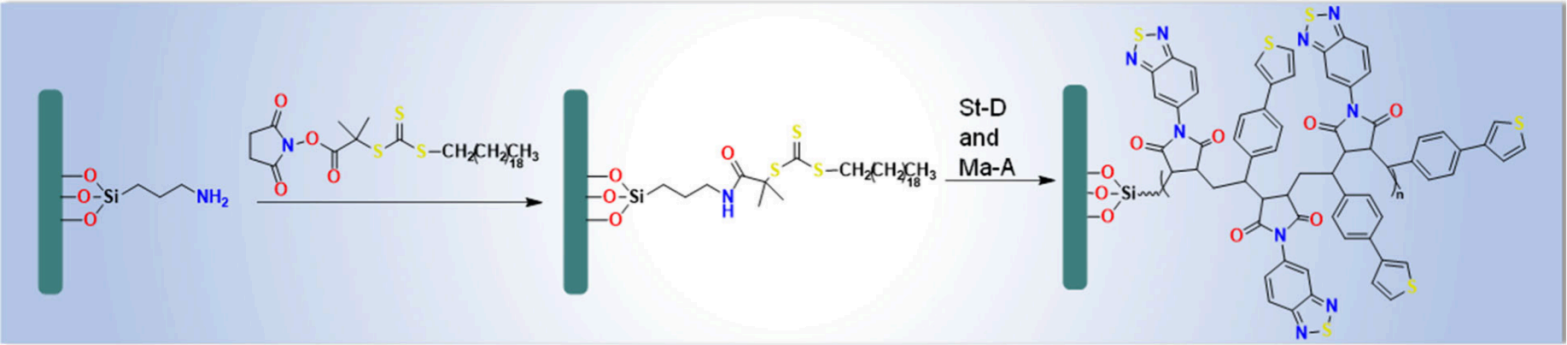
Synthesis of donor–acceptor poly(St-*D*-*alt*-Ma-A) brushes via RAFT polymerization. Adopted with permission under Creative Commons CC BY 4.0 license from ref (99). Copyright 2022 The Author(s).

The process used to synthesize D–A polymers should allow for the possibility of alternate monomer arrangements along the chain as shown in (Figure 11). Because of this, the range of approaches that are currently accessible is somewhat restricted to direct arylation^93,94^ or catalyzed cross-coupling reactions,^95,96^ which are carried out in accordance with Stille,^97,98^ Suzuki,^33−36^ and Sonogashira,^41−44^ processes. They all have rather strict requirements, which limits the efficiency of the finished items and occasionally necessitates a laborious multistep process. In addition to meeting all specifications needed to produce conjugated chains, the synthesis process for alternating D–A polymer brushes also needs to guarantee an alternate arrangement of monomers along the chain. In 2022 Grobelny et al.^99^ reported a proof of concept for a synthetic process utilizing surface-initiated reversible-deactivation radical polymerization of the alternatingly polymerizable monomers to produce alternating D–A PB. Because styrene and maleic anhydride derivatives are known to exhibit an alternating copolymerization tendency, they were employed. After attaching thiophene (an electron donor) and benzothiadiazole (an electron acceptor) to styrene and maleic anhydride, respectively, surface-initiated RAFT polymerization and metal-free ATRP were used to create the copolymer brushes.

### 4.2 Synthesis via Living Anionic Polymerization

**Figure 12:**
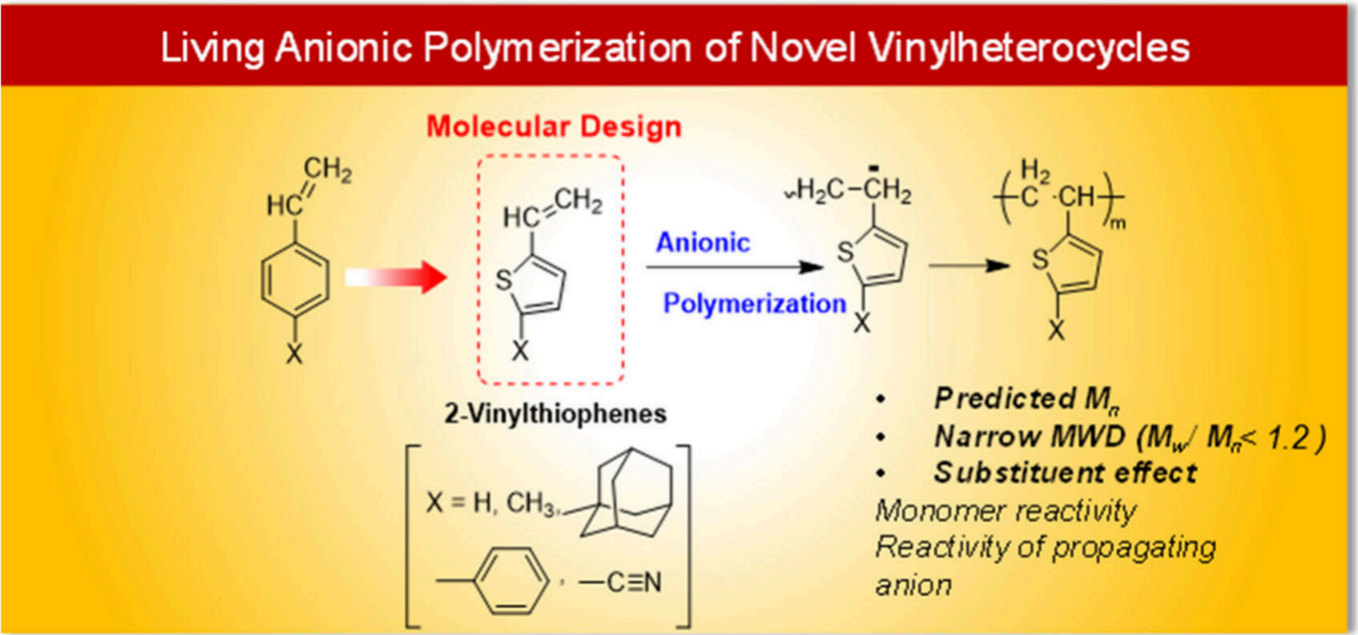
Living anionic polymerization of 5-substituted 2-vinylthiophenes. Adopted with permission under Creative Commons CC BY 4.0 license from ref (100). Copyright 2024 The Authors.

The most well-established technique for creating well-defined polymers with expected molecular weights and narrow molecular weight distributions (MWD) is the live anionic polymerization of vinyl monomers. The living characteristics of the propagating species can be used to manufacture both end-functionalized polymers and a variety of architectural polymers, such as block copolymers, graft polymers, and star-shaped polymers. In 2024 Kurishiba et al.^100^ presented findings about the anionic polymerization of various 2VTs, such as nonsubstituted 2-vinylthiophene (1) (Figure 12). To disclose the electronic and steric effects on the anionic polymerizability of 2VTs and the reactivity of the carbanion that 2VTs formed, substitutes such as methyl (2), 1-adamantyl (3), phenyl (4), and cyano (5) groups were added.

### 4.3 Synthesis via Cationic Polymerization

**Figure 13:**
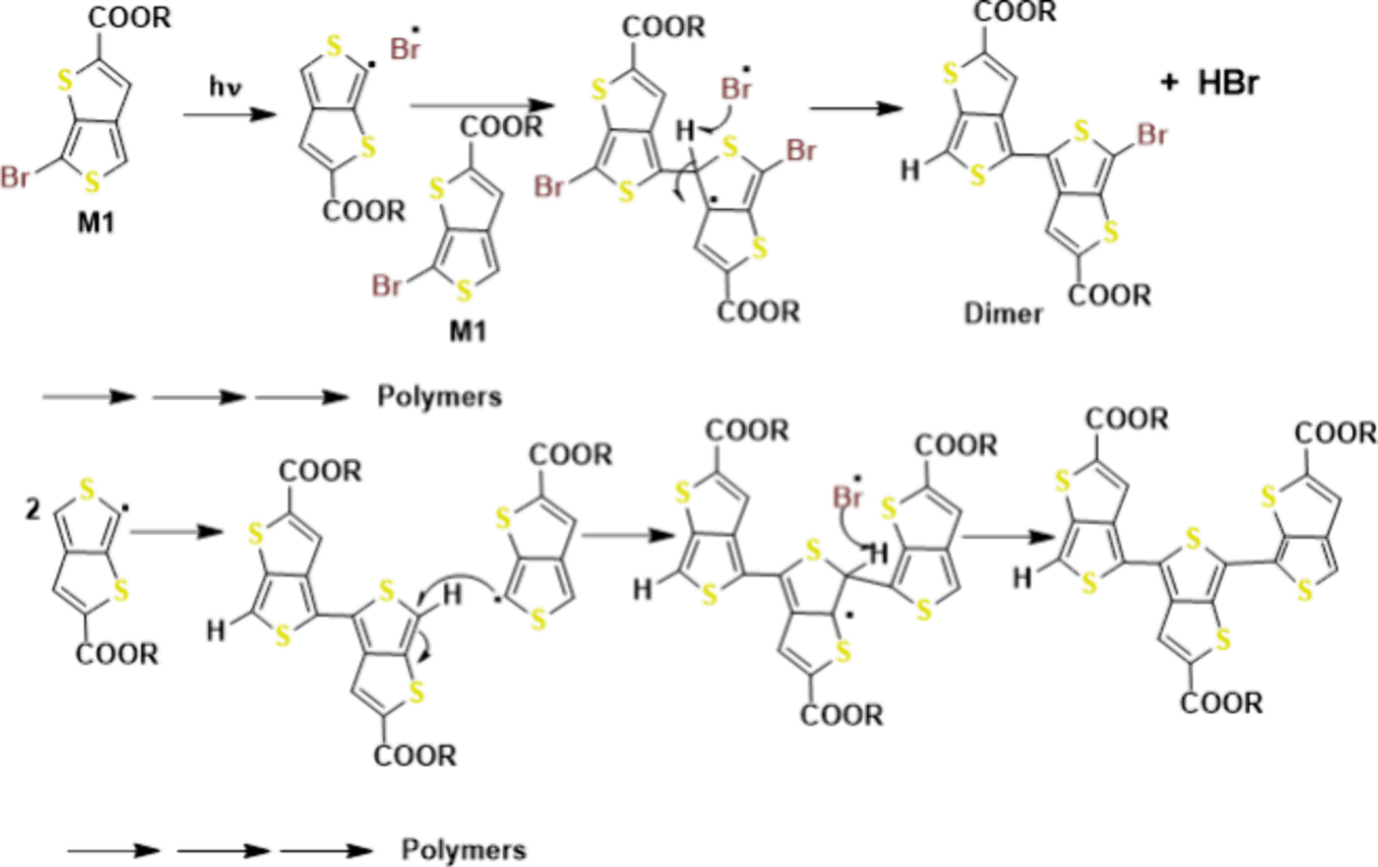
Proposed polymerization mechanism. Adopted with permission from ref (101). Copyright 2020 Royal Society of Chemistry.

Living cationic polymerization is one such technique, known for its ability to produce polymers with a narrow molecular weight distribution and controlled architecture as shown in (Figure 13). This process is particularly advantageous for synthesizing conjugated polymers where control over polymer size and end groups is crucial for performance in electronic applications. In 2020 Liu et al.^101^ reported an accidental discovery of a photoinduced solid state polymerization of monobromo-thieno[3,4-*b*]thiophene (M1) to generate NIR conjugated polymers with an ultralow optical band gap. Their results had substantial ramifications in both synthesis of conjugated polymers and the mechanism for cationic chain-growth polymerizations utilizing aromatic monomers.

### 4.4 Synthesis via Atom Transfer Radical Polymerization

**Figure 14:**
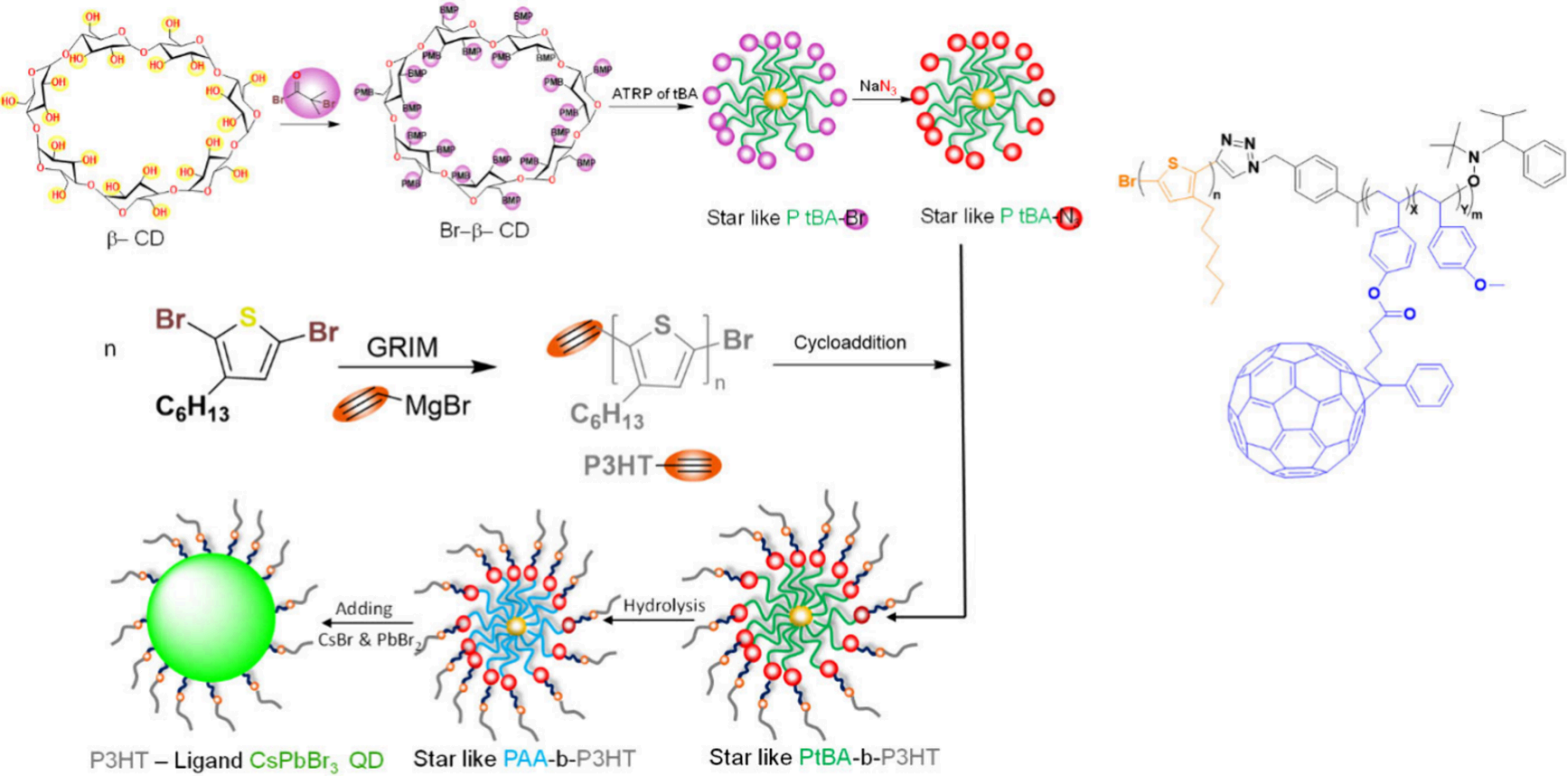
Synthesis of P3HT-ligated CsPbBr_3_ QDs. Schematic representation of crafting CsPbBr_3_ QD via capitalizing on starlike PAA-*b*-P3HT block copolymer as a nanoreactor. Adopted with permission from ref (107). Copyright 2022 American Chemical Society.

Atom Transfer Radical Polymerization (ATRP) is a controlled/living radical polymerization technique that allows for the synthesis of polymers with well-defined molecular weights and narrow molecular weight distributions. ATRP is particularly useful for synthesizing conjugated polymers, which are polymers with alternating single and double bonds along their backbone, crucial for applications in organic electronics, sensors, and photonic devices.^102−104^ The synthesis of well-defined r poly(3-hexylthiophene) (P3HT) based rod–coil diblock copolymers (*Đ* ≤ 1.3) in good yield and high purity reported in 2018 by Nguyen et al.^105,106^ P3HT was the macroinitiator for the UV light-induced metal-free ATRP of different methacrylate monomers. In order to develop P3HT-based copolymers, pyrene and a novel phenothiazine derivative (10-(pyren-1-yl)-10H-phenothiazine (PPTh)) were studied as photocatalysts. For the first time, in 2022 Liang et al.^107^ reported staggered type-II band alignment could be achieved by precisely controlling the length of CPs and the size of perovskite QDs in CP-ligated, monodisperse perovskite QDs, thereby tailoring charge separation at a series of painstakingly engineered CP/perovskite QD interfaces. (Figure 14)

### 4.5 Synthesis via Nitroxide-Mediated Radical Polymerization (NMP)

**Figure 15:**
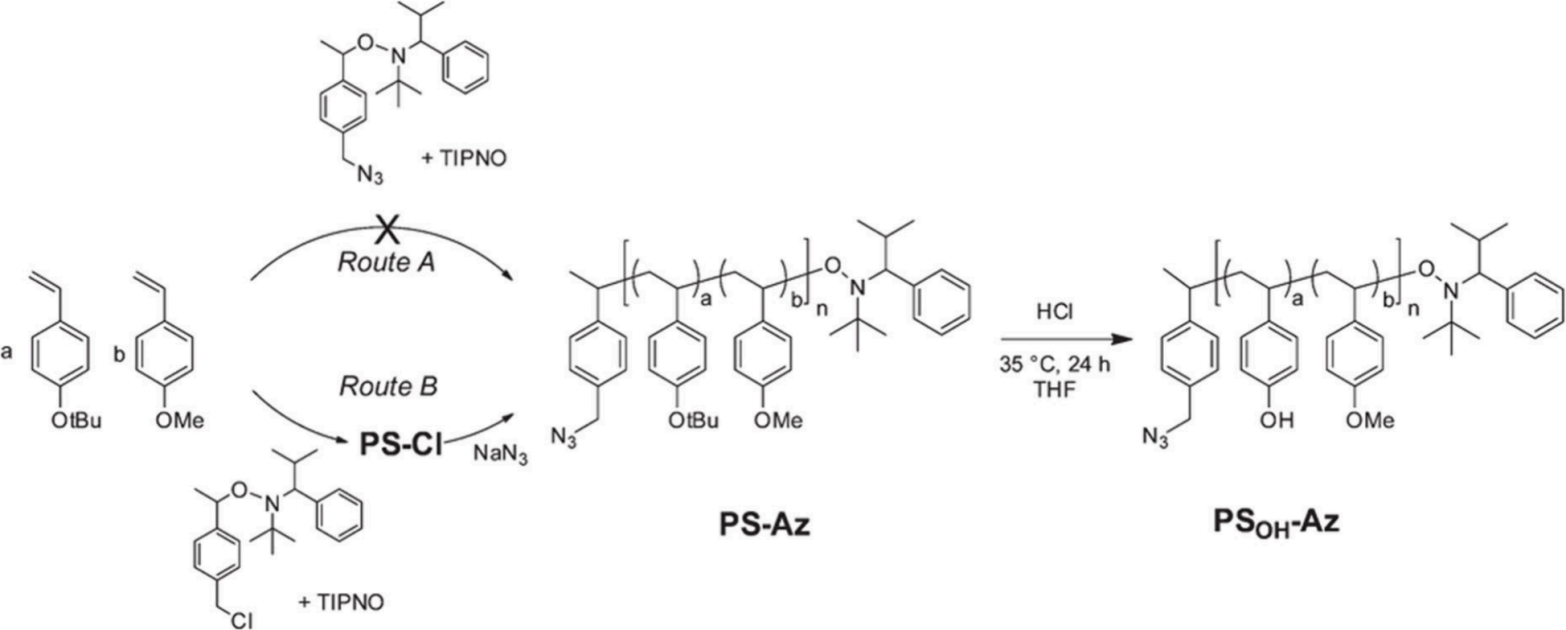
Synthesis strategy toward the azide-terminated precursor copolymer poly(4-methoxystyrene-*stat*-4-hydroxystyrene). Introduction of the azide end group is attempted by two different methods, depicted as routes A and B. The hydroxyl functionality is achieved by hydrolysis of the *tert*-butyl ether groups in PS-Az maintaining the azide end group to obtain PSOH-Az. Adopted with permission from ref (105). Copyright 2015 WILEY-VCH Verlag GmbH & Co. KGaA, Weinheim.

Nitroxide-mediated polymerization (NMP) is a reversible-deactivation radical polymerization (RDRP) technology that allows for the creation of well-defined and complex macromolecular structures with low polymer dispersion and excellent chain end homogeneity.^108,109^ Poly(3-hexylthiophene) P3HT and other conjugated polymers have a tendency to be stiff and rod-like, whereas polymers synthesized using NMP have a flexible backbone and behave more like a regular coil as reveal in. Both the well-defined coil–coil block copolymer produced by NMP and the rod–coil, in which the rod is produced using a different method like Grignard metatheses (GRIM), have been used by researchers. When compared to using blends of the respective homopolymers, Thelekkat and colleagues’ usage of NMP to construct coil–coil triaryl amine (donor) and perylene bisimide (acceptor) based block copolymers showed enhanced OPV performance in the early 2000s. A statistical copolymer of poly(4methoxystyrene-*stat*-4-tert-butoxystyrene) was recently synthesized by Thelakkat et al.^108^ using NMP (Figure 15). PCBM was grafted by an esterification reaction after a P3HT block’s click reaction, resulting in a rod–coil P3HTfullernee based block copolymer.

## 5 Properties of Smart Multistimuli-Responsive Conjugated Polymer Materials

### 5.1 Optical Properties

**Figure 16:**
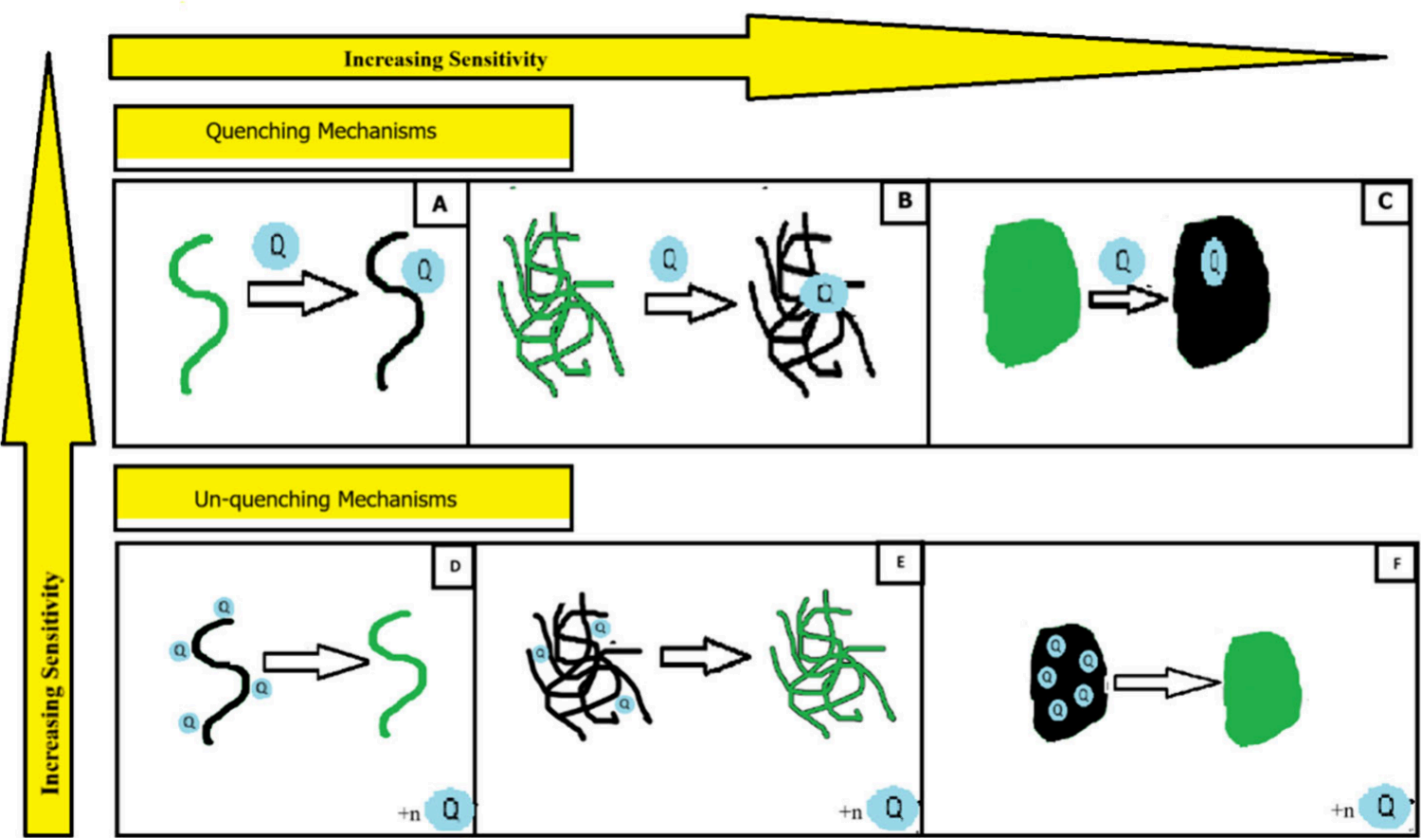
Effect of dimensionality on (un)quenching mechanisms. Quenching mechanisms are by nature more sensitive than unquenching mechanisms (a single quencher can affect the properties of a larger fluorophore), whereas higher-dimension materials display higher sensitivity relative to one-dimensional, or isolated, polymer wires. Adopted with permission from ref (115). Copyright 2013 American Chemical Society.

Synthesized Conjugated polymers show various optical properties.^110,111^ In particular, stimuli-responsive π-conjugated polymers have attracted due to their unique optical and electrical properties, which are useful in applications such as light-emitting diodes,^112^ solar cells,^113^ and biosensors.^114^ Due to rising social and industrial needs, novel materials with distinct functionalities and qualities have recently received a lot of attention. Specifically, a multiplicity of sensory systems and transduction mechanisms were developed by their application of sensing schemes and devices, which was the subject of extensive investigation. Conjugated polymers are especially well-suited for a wide range of sensing applications due to their many appealing properties. In (Figure 16) exhibit how these ideas can be applied to two distinct signal transduction modalities. The addition of an analyte that quenches the polymer’s excited state by energy or electron transfer is part of the quenching mechanism. In this process, the material’s shape and exciton diffusion length determine the gain; continuous films exhibit the best amplification.

### 5.2 Biointerface

**Figure 17:**
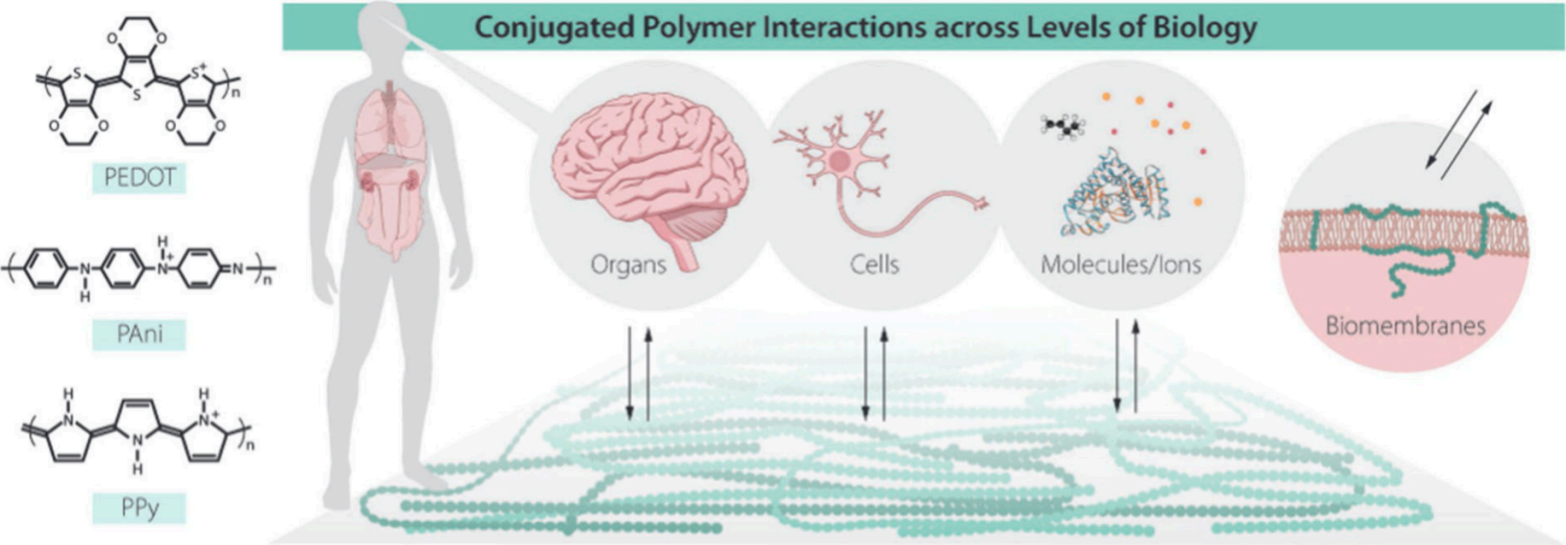
Conjugated polymer materials can interact with biology at different hierarchical levels ranging from organs and tissues down to molecules and ions. Left: Three examples of commonly used conjugated polymers. From top to bottom: poly(3,4-ethylenedioxythiophene), poly(aniline), and poly(pyrrole). Adopted with permission from (120). Copyright 2019 WILEY-VCH Verlag GmbH & Co. KGaA, Weinheim.

The need for materials that can interface with biological materials is growing as biomedical engineering advances, particularly for applications such as biosensors, medical implants, brain interfaces, and scaffolds for tissue engineering. Due to their superior mechanical qualities, improved biocompatibility, and intrinsic conductivity when compared to traditional metal-based materials, conducting polymers are intriguing alternatives for these applications. These benefits can be further enhanced by functionalizing the conducting polymers, which will provide qualities like better solubility, antifouling behavior, stimuli-responsive switchability, and the capacity to control cell division and growth. Since it was shown that many CPs are biocompatible and may be used to electrically stimulate cells, changing cellular processes, research into employing CPs as biointerfaces has increased significantly.^116,117^ Mainly, nerve, bone, and muscle cells—cells known to respond to electrical stimulation in vivo—have been used to investigate electrical stimulation.^118^ However, CPs need to be further functionalized using biomolecules or biocompatible polymers because they do not interact with cells and tissues in an optimal manner. Thus, the development of technologies to functionalize CPs opens up tremendous prospects to generate materials more suited for various biointerface applications.^116^ Because of their distinctive electric and optical properties, as well as their polymeric nature and biocompatibility, conducting polymers have drawn a lot of interest as biointerfaces.^119^ CPs are appealing candidates for a variety of biomaterials because of their low cost, ease of production, and outstanding biocompatibility, especially in applications that need communication across interfaces. For example, CPs have a wide range of applications as (bio)sensors because of their ability to transduce chemical signals into optical or electronic signals. Professor Olle Inganäs’^120^ contributions to the subject of CPs for monitoring and managing biological activities, as well as his most relevant publications on each issue, are highlighted. His research covered the use of these conjugated organic materials at all biological levels, as shown in (Figure 17) bulk/surface interactions with organs and tissues, down to cells, and at the most fundamental level—to which the others can be traced back—with molecules and ions, as well as interactions of individual bioelectronic molecules inserted into biomembranes.

### 5.3 Antimicrobial Properties of Conjugate Polymers

**Figure 18:**
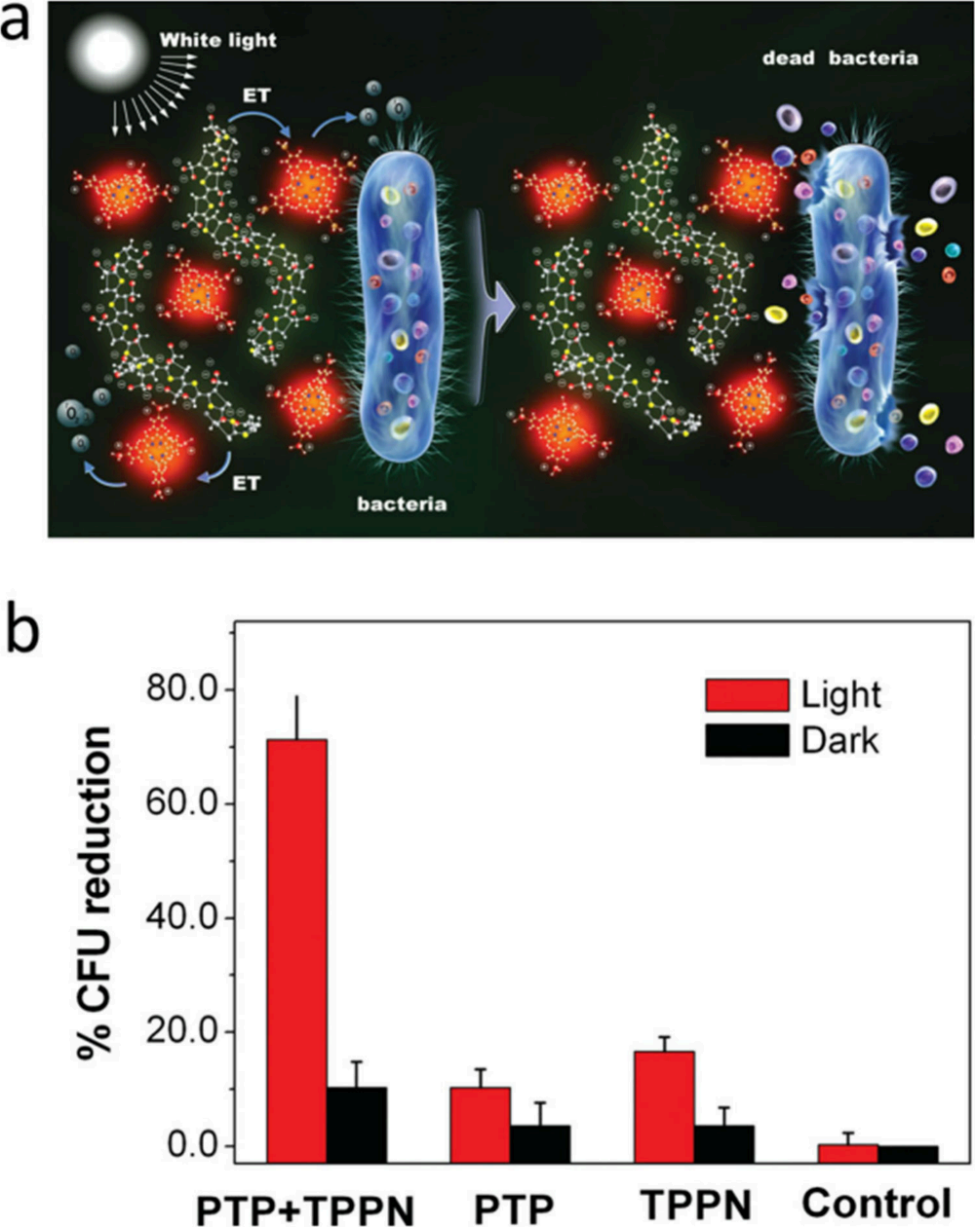
(a) Schematic antibacterial mechanism of P5/TPPN complex. (b) Biocidal activity of P5/TPPN, P5, and TPPN toward *E. coli* in the dark and under white-light illumination for 5 min at a fluence rate of 90 mW cm^–2^. Adopted with permission from ref (124). Copyright 2009 American Chemical Society.

CPs’ excellent light-harvesting capacity and effective energy transfer have piqued interest in them as promising materials for photodynamic treatment (PDT). They have a huge π-delocalized electronic system that allows the exciton to hop between chains or travel along the polymer backbone chains. Photosensitizers (PSs) and other dye acceptors can transfer energy efficiently because of the exciton’s capacity to spread within CP chains.^121^ Additionally, CPs can directly sensitize oxygen molecules to form reactive oxygen species (ROS) following photoexcitation. ROS have the potential to seriously harm biomacromolecules used in antibacterial and anticancer applications.^122^ With the application of visible light, Whitten and colleagues discovered a cationic conjugated polyelectrolyte derivative that is capable of killing both Gram-positive and Gram-negative bacteria.^123^ This groundbreaking work established a new area of CP research in photodynamic therapy. Since then, a number of hydrophobic CPNs and conjugated polyelectrolytes have shown their usefulness for PDT. With reference to the biological and medicinal uses of CPNs, the Wang group has thoroughly examined the synthesis of CPNs for drug delivery and release, gene delivery, fluorescence imaging, and antimicrobial and anticancer properties. Since then, many families of CPNs have been successfully and quickly created from polymer from design to use. In addition to PDT, CPNs are a new class of antibacterial substance that exhibits exceptional activity and is thought to be a strong contender to overcome bacterial resistance that is more effective due to two distinct mechanisms of action, beneath both in the light and in the dark. In 2009 Wang et al.^124^ reported on a novel CP-based energy-transfer system designed to eradicate germs.. An electrostatic compound was generated by the cationic porphyrin (TPPN) and anionic PT. Effective energy transfer from P5 to TPPN (Figure 18a) increased the singlet oxygen generation rate under white-light irradiation compared to TPPN’s own rate (Figure 18a). Because of electrostatic and hydrophobic interactions, the P5/TPPN (Figure 18a) complex may attach to the Gram-positive *Bacillus subtilis* and Gram-negative *Escherichia coli* bacteria. The P5/TPPN energy-transfer system demonstrated significant efficacious biocidal activity against these bacteria at low light doses (Figure 18b).

### 5.4 Mechanical Properties

Stimuli-responsive MACPs can also exhibit changes in mechanical properties: Some MACPs can undergo reversible mechanical deformations in response to external stimuli, useful in soft robotics and adaptive materials.^125^ The incorporation of flexible acceptor units can enhance the polymer’s elasticity and flexibility, making them suitable for flexible electronics and wearable devices. When combined with the mechanical characteristics common to macromolecules, conjugated polymers display a unique range of electrical and electrochemical behavior that makes them attractive candidates for a variety of application areas, from wearable electronics to bioelectronics.^126,127^ However, when building flexible or stretchable devices, the degree of oxidation or reduction of the polymer must be taken into account since it can significantly effect the mechanical response. The scaling laws that apply to other forms of polymers also apply to conjugated polymers. If the relaxation period is long enough, polymeric materials can dissipate stress through conformational changes that can take place on a variety of length scales, from the size of single functional groups, side chains, or repeat units to whole polymer chains. This results in a complicated viscoelastic behavior whose mechanical and rheological properties depend on temperature as well as on time, frequency, and rate.^128,129^

## 6 Applications of Multiacceptor Conjugated Polymers

### 6.1 Optoelectronics Device

**Figure 19:**
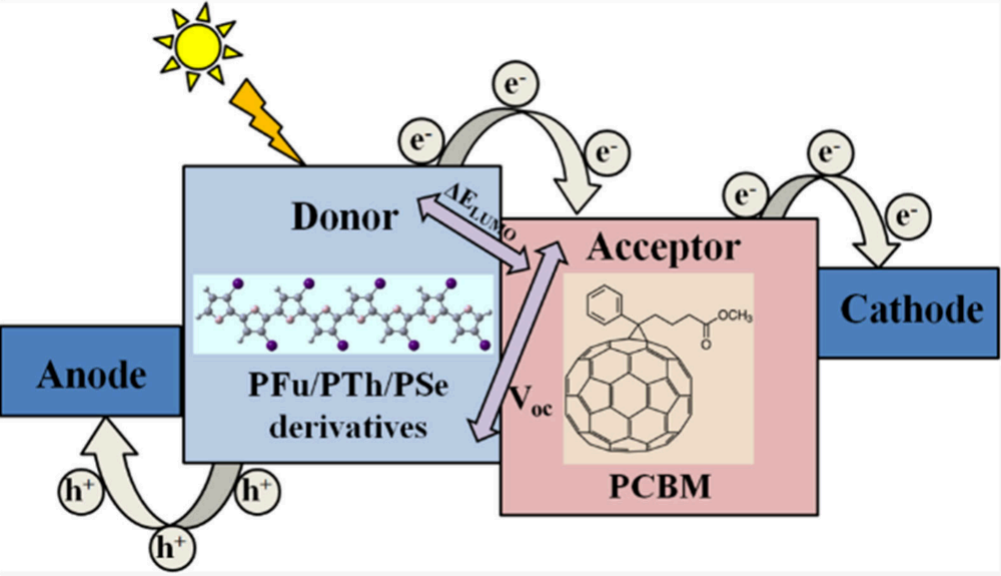
Schematic diagram of optoelectronic applications of conjugated organic polymers. Adopted with permission from ref (138). Copyright 2022 American Chemical Society.

The frontier molecular orbitals (FMOs) of PPV devices must be precisely regulated in order to increase their efficiency. Because they possess an adjustable band gap (Egap), which is necessary for high conductivity, high nonlinear optical sensitivity, and potential transparency in the visible portion of the absorption spectrum, π-conjugated organic polymers (COPs) are the perfect materials to develop efficient photovoltaic devices.^111,130^ The organic polymer chain’s ability to transfer charge is facilitated by the π-conjugation, which results from alternating single and double carbon bonds.^131^ The aforementioned polymers exhibit either conducting or semiconducting behavior. They possess the capability to be incorporated into optical and electronic devices, including organic polymer-based transistors, photoresistances, electrochromic devices, light-emitting diodes (LEDs),^132−134^ and OSCs.^135^ By chemically modifying them (introducing side groups into the polymer chain) or by doping them, it is possible to tune their Egap and increase the absorption spectrum’s width by. This allows for their implementation in photovoltaic devices. Optoelectronics uses functionalized conducting polymers, according to a review by Khokhar et al.^136^ Ullah et al.^137^ have studied polypyrrole doping and dedoping. In 2022, Garg et al.^138^ and reported the impact of distinct side groups that release and withdraw electrons on the structural and electrical characteristics of polymers. The objective was to create a polymer that would improve the open circuit voltage (VOC) and short circuit current density (JSC) for application in BHJ solar cells. (Figure 19)

### 6.2 Sensors and Actuators

**Figure 20:**
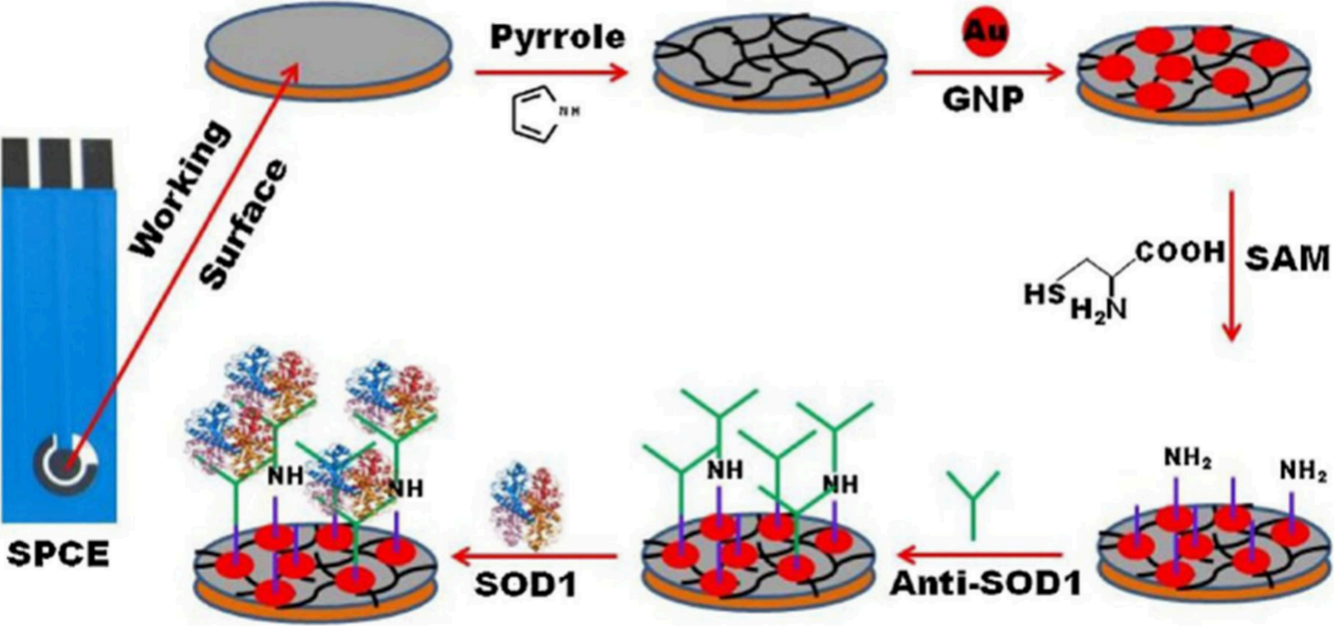
Free electrochemical immunosensor for the rapid and sensitive detection of the oxidative stress marker superoxide dismutase 1 at the point-of-care. Adopted with permission from ref (142) Copyright 2016 Elsevier B.V.

MACPs are used in a variety of sensor and actuator applications due to Conducting polymers (CPs) including semiconducting molecular structures and interesting features that are well-suited for sensing applications because of the electronic conjugation between each repeat unit. Therefore, sensor designs utilizing CPs are shown to have made significant development.^139−141^ Unfortunately, using pure CPs makes it difficult to meet the most fundamental needs of sensors, such as analyte selectivity and the ability to detect a particular analyte in a complicated environment. Superoxide dismutase (SOD1) can be detected using a label-free electrochemical immunosensor that was created by modifying the screen-printed electrode (SPE) and bio functionalizing electro synthesized polypropylene (PPy) with a monoclonal antibody (anti-SOD1). The SOD1 immunosensor antiSOD1/SAM/GNP/PPy/SPCE demonstrated a high degree of SOD1 detection within nanomolar and micromolar concentrations, which are typically present in plasma, serum, and blood as well as the cytosol of neuronal cells.^142^ (Figure 20)

### 6.3 Bioelectronics

**Figure 21:**
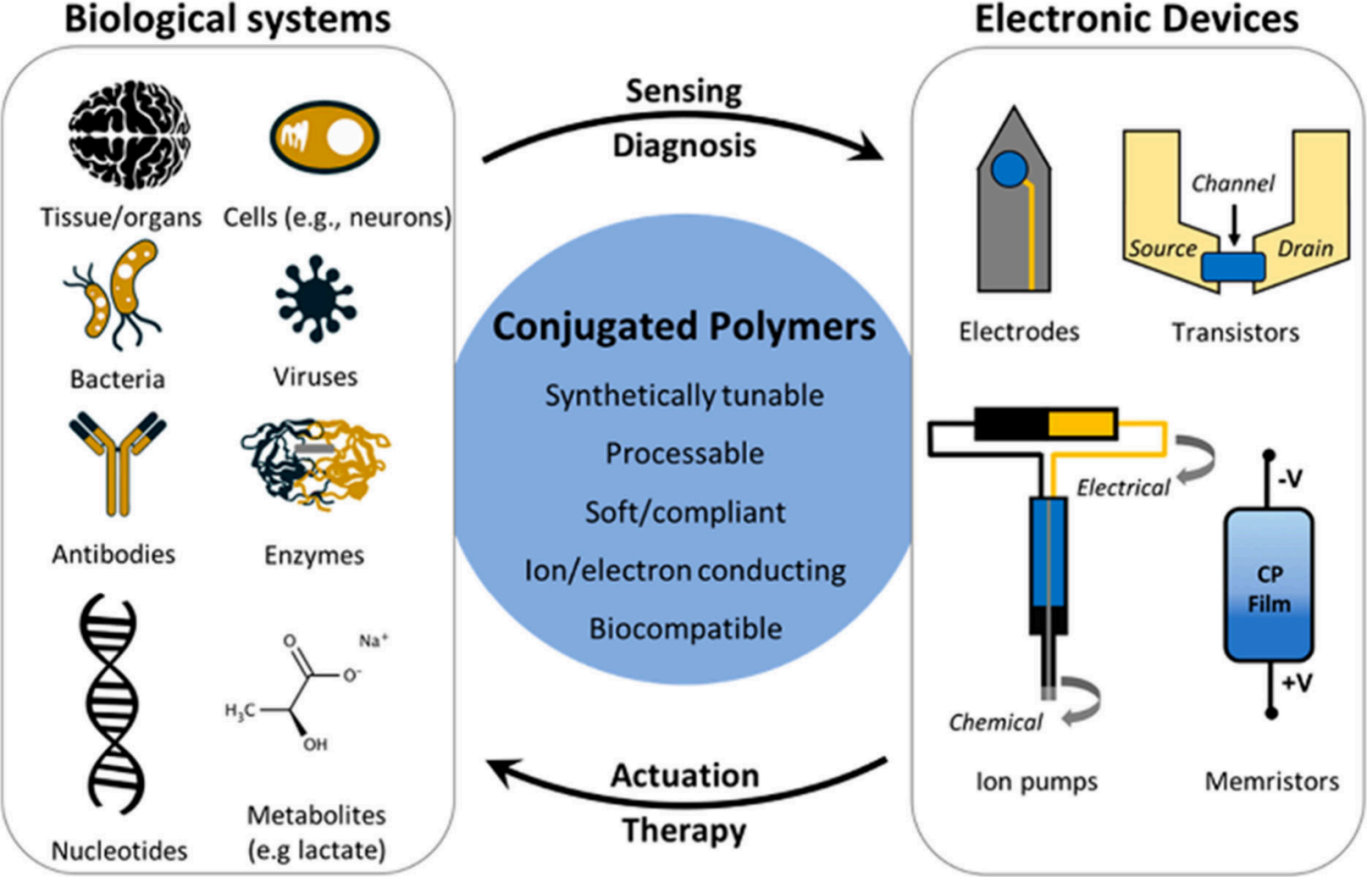
Unique set of properties of conjugated polymers that lead to state-of-the-art devices used for sensing or actuation. Adopted with permission from (146). Copyright 2018 American Chemical Society.

The field of organic bioelectronics is a young one that aims to connect the soft, mostly ionic realm of biology with the electrical realm of organic semiconductor-based devices. Crosstalk like this might happen both ways.^20,143^ An organic material’s doping state could be altered by a biological event, for instance, producing an electrical readout. On the other hand, a biological event might be triggered by an electronic signal from a gadget. Innovative studies in this domain lead to the creation of numerous useful applications, ranging from brain-machine interfaces and health monitoring tools to biosensors and medication delivery systems. Conjugated polymers can be created into a variety of shapes and sizes, such as hydrogels with ionically conducting properties and Young’s moduli akin to those of soft tissues.^144^ They also share chemical “nature” similarities with biological molecules. Synthetic chemistry can be used to modify the structure of organic materials, and different functionalization techniques can be employed to regulate their biological characteristics. Ultimately, devices with form factors that facilitate integration with biological systems can be created by integrating organic electronic materials with a range of mechanical supports. Although these advancements are novel and encouraging, it is crucial to remember that the subject is still in its infancy, with a great deal of unanswered questions and a vast amount of room for highly collaborative study and discovery.^145^ (Figure 21)

### 6.4 Biomedical Applications

**Figure 22:**
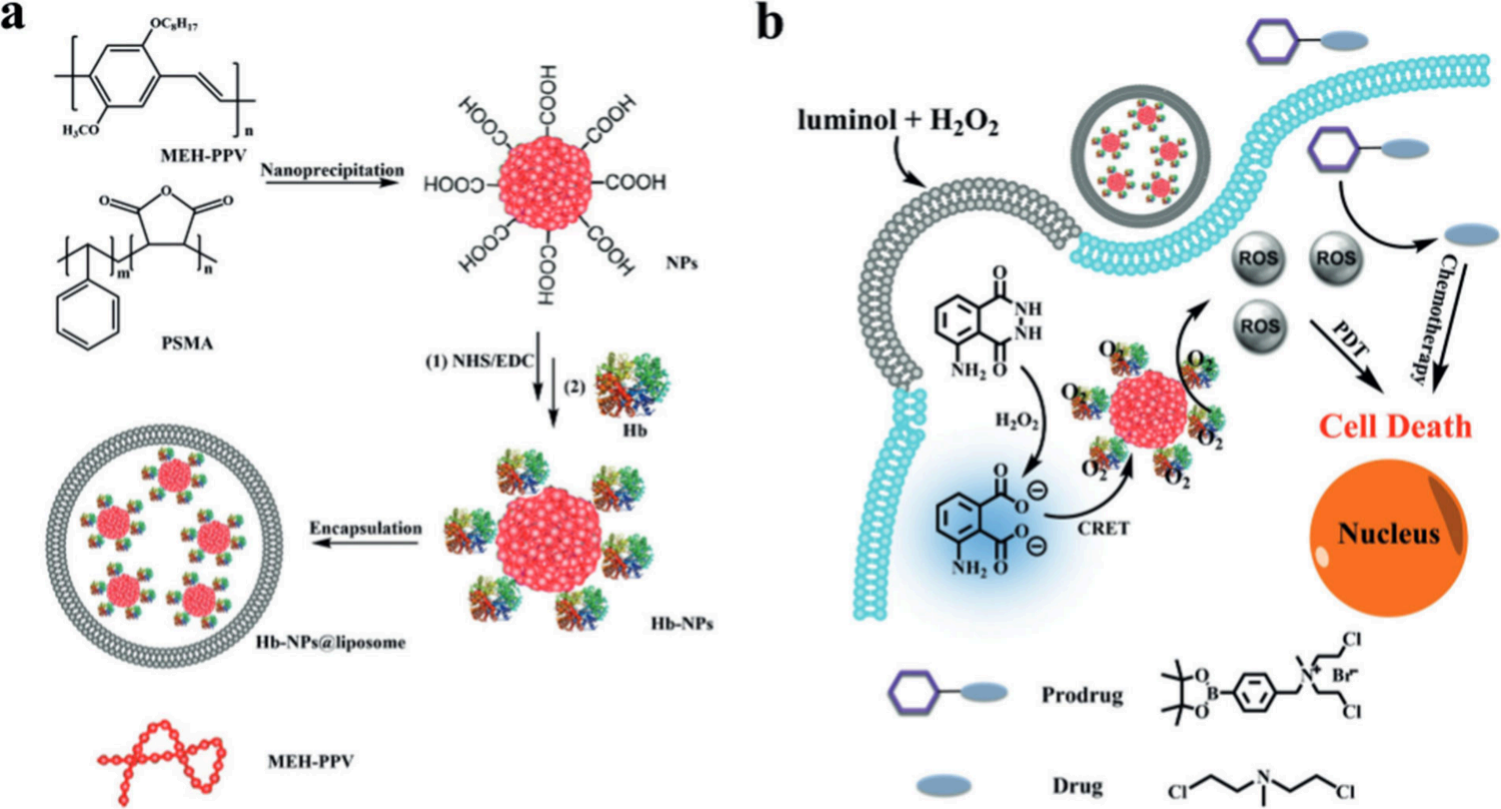
(a) Preparation process of Hb-NPs@liposome. (b) Schematic illustration of BL and the oxygen-supplying system for photodynamic therapy. Adopted with permission from ref (150). Copyright 2019 Wiley-VCH Verlag GmbH & Co. KGaA, Weinheim.

Large π-conjugated backbones and delocalized electronic structures characterize conjugated polymers (CPs), a class of organic semiconductor materials. Numerous CPs with different chemical structures and functions are rapidly developing in a wide range of biomedical fields, including fluorescence imaging, photodynamic therapy, photothermal therapy, etc., because of their unique photophysical properties and photoelectric effects.^147–149^ Furthermore, the predicted biological response, biocompatibility, water solubility, and other properties may be facilitated by the functionalized side chains of CPs. By using the common synthesis technique, CPs may also be synthesized into nanoparticles for controlled particle size and dispersion. Wang and colleagues developed a novel CL system based on hemoglobin (Hb) and CPNs to significantly raise the oxygen level in the hypoxic microenvironment of the solid tumor.^150^ The first step involved coencapsulating conjugated polymer poly[2-methoxy-5-(2-ethylhexyloxy)-1,4-phenylenevinylene] (MEH-PPV) and poly(styrene-*co*-maleicanhydride) (PSMA) into nanoparticles (NPs) with carboxyl groups on the surface using the nanoprecipitation method, as depicted in (Figure 22a,b).

## 7 Challenges and Future Directions

A variety of strategies have been explored to improve the end functionalities and molecular architectures of conjugated polymers to make them suitable for various applications, as described in several reviews or book chapters. Heinze et al.^17^ for instance, detailed the significant advancements made in the field of CP electrochemistry during the past few years. Beginning with the electrochemical creation mechanism of CPs, it has been made clear that oligomerization happens in solution in front of the electrode and is best based on ionic (radicalic) species that go through successive “dimerization” phases. However, the topic of CPs’ multiacceptor system was left out. Schroot et al.^151^ reported building blocks to illustrate the simple assembly to Dn–P–Am architectures—which effectively block copolymers with a single interjacent photosensitizer unit—were described as telechelic redox-active and conjugated polymers. This article has not covered the multiacceptor system in CPs. Nezakati et al.^19^ reported the opportunity of conducting polymer and biomedical applications. CPs and their advantages and associated challenges, from synthesis to applications, were fully discussed but there is no multiacceptor system described. Inal et. al^152^ described conjugated polymers in bioelectronics but multiacceptor system in CPs were not covered. All these reviews mainly focused on the synthesis of conjugated polymers via different strategies and their potential applications. However, there is no comprehensive Review in the past 10 years (and before) Notably, multistimuli responsive multiacceptor DA in conjugated polymers have never been reviewed (in contrast to other conjugated polymers) which have been reviewed extensively. Moreover, none of the reviews mentioned in this section focused on the aspects and uniqueness of stimuli-responsive multiacceptor DA in conjugated polymers which may introduce smart properties in such conjugated polymers.

## 8 Conclusions

Stimuli-responsive conjugated polymers represent a promising frontier in materials science, offering unique capabilities to respond to external triggers such as light, temperature, pH, and electric fields. These polymers exhibit changes in their optical, electrical, or mechanical properties in response to stimuli, making them highly versatile for applications in fields like sensors, drug delivery, and flexible electronics. The ability to tune their properties through chemical modification and structural design allows for the creation of smart materials that can adapt to their environment. Current research has demonstrated significant advances in understanding the mechanisms behind their responsiveness, leading to improved performance in practical applications. Developing polymers that can respond to multiple stimuli simultaneously will enhance their functionality, allowing for more complex applications in smart devices. Future research should focus on designing polymers that are biocompatible and environmentally friendly, facilitating their use in biomedical applications without adverse effects. Integrating self-healing capabilities into conjugated polymers could significantly enhance their durability and lifespan, making them suitable for applications in wearable electronics and structural materials. Focusing on green chemistry and sustainable practices in the synthesis of these polymers will be crucial in addressing environmental concerns associated with traditional manufacturing methods. By pursuing these directions, the field of stimuli-responsive conjugated polymers can continue to evolve, leading to innovative solutions that integrate seamlessly into technology and everyday life.
